# Not so simple, not so subtle: the interspecies competition between *Bacillus simplex* and *Bacillus subtilis* and its impact on the evolution of biofilms

**DOI:** 10.1038/npjbiofilms.2015.27

**Published:** 2016-01-27

**Authors:** Gili Rosenberg, Nitai Steinberg, Yaara Oppenheimer-Shaanan, Tsvia Olender, Shany Doron, Julius Ben-Ari, Alexandra Sirota-Madi, Zohar Bloom-Ackermann, Ilana Kolodkin-Gal

**Affiliations:** 1 Department of Molecular Genetics, Weizmann Institute of Science, Rehovot, Israel; 2 The Laboratory for the Mass Spectrometry and Chromatography, The Interdepartmental Analytical Unit, The Robert H. Smith Faculty of Agriculture, Food and Environment, the Hebrew University of Jerusalem, Rehovot, Israel; 3 Department of Biostatistics, Harvard School of Public Health, Boston, MA, USA

## Abstract

*Bacillus subtilis* biofilms have a fundamental role in shaping the soil ecosystem. During this process, they unavoidably interact with neighbour bacterial species. We studied the interspecies interactions between biofilms of the soil-residing bacteria *B. subtilis* and related *Bacillus* species. We found that proximity between the biofilms triggered recruitment of motile *B. subtilis* cells, which engulfed the competing *Bacillus simplex* colony. Upon interaction, *B. subtilis* secreted surfactin and cannibalism toxins, at concentrations that were inert to *B. subtilis* itself, which eliminated the *B. simplex* colony, as well as colonies of *Bacillus toyonensis*. Surfactin toxicity was correlated with the presence of short carbon-tail length isomers, and synergistic with the cannibalism toxins. Importantly, during biofilm development and interspecies interactions a subpopulation in *B. subtilis* biofilm lost its native plasmid, leading to increased virulence against the competing *Bacillus* species. Overall, these findings indicate that genetic programs and traits that have little effect on biofilm development when each species is grown in isolation have a dramatic impact when different bacterial species interact.

## Introduction

Soil bacteria have a central role in shaping soil ecology. The soil microorganismic community is composed of diverse populations of bacterial species that dramatically affect the availability of soil nutrients and plant diversity.^1–4^ The immense bacterial diversity within the soil leads to unavoidable interspecies interactions, which ultimately form a structured microbial community.^5,6^ Generation of antagonistic and mutualistic behaviours, mediated by exchange of small diffusible secondary metabolites, enables bacterial adaptation to the complex communal life.^6,7^ Such communication can induce resistance to various antibiotics, or can eliminate rival bacterial species competing for limited nutrients.

Most bacteria in nature live in structurally and dynamically complex biological systems called biofilms. Biofilms are multicellular communities of surface-associated bacteria enveloped in a self-produced extracellular matrix.^8^ The extracellular matrix isolates the bacteria in the biofilm from the external environment and protects them from antibiotics, sterilising agents and the immune system.^9^ Here we studied the interactions between two robust biofilm formers—*Bacillus subtilis* and the closely related *Bacillus simplex.* Both bacterial species reside in and compete for the same ecological niche—the soil.^10,11^

*B. subtilis* is a master of differentiation, displaying a multitude of distinct cell types within its biofilms. *B. subtilis* can differentiate into cells capable of taking up DNA from the environment.^12–14^ During the development of genetic competence, the production of a small cyclic lipopeptide named surfactin is induced.^15^ The machinery for surfactin synthesis is encoded within the *srfAA-AB-AC-AD* operon. Surfactin is also a powerful surfactant that shows a wide range of biological activities such as antibacterial,^16^ antiviral and antifungal actions.^17^ It is composed of an amphipathic, cyclic heptapeptide head group that is interlinked with a hydrophobic β-hydroxy fatty acid tail, comprising 12–16 carbon atoms.^18–20^ These features enable the surfactin molecule to act on cellular membranes and disrupt the membrane integrity.^21^ The production of surfactin is sensed together with additional environmental signals, by a portion of the biofilm population that then produces the extracellular matrix.^8,22,23^ In addition, this subpopulation produces an extracellular killing factor SkfA and SdpC that function to kill (or cannibalise) cells that have not yet commenced to sporulation.^24^ Importantly, the production of cannibalism toxins was only contributing to biofilm development in mutants where genes predicted to have a role either in the production of, or in the sensitivity to, cannibalism toxins were deleted.^25^ Thus, it is still unclear, what is the exact function of the cannibal cells in wild-type (WT) biofilms. In a biofilm, an additional distinct subpopulation of cells express *sigD*, the sigma factor necessary for flagella production, resulting in motile heterogeneity.^26^ The role of motile cells in biofilms that are formed over solid surfaces remains unknown.

The main components of the *B. subtilis* extracellular matrix are exopolysaccharides, synthesised by the *epsA-O* operon-encoded genes, TasA, a functional amyloid, encoded in the three-gene operon *yqxM\TapA-sipW-tasA*^27,28^ and BslA, that forms a hydrophobic coat over the biofilm.^29^ The master regulator controlling the switch to a biofilm lifestyle is the repressor SinR.^30^ The commitment to the biofilm state requires the phosphorylation of Spo0A. Spo0A-P activates SinI, which directly binds and represses SinR.^31^ Recently, an additional regulatory gene for biofilm formation, *rapP*, was found on the native 80-kb pBS32 plasmid, which was lost during *B. subtilis* domestication.^32^ RapP encodes a phosphatase that dephosphorylates the intermediate response regulator Spo0F, and thus indirectly represses Spo0A activity.^33^ The decreased phosphorylation of Spo0A results in an altered biofilm formation.^32,33^ In addition, RapP regulates genetic competence.^34^

Here, we demonstrate that upon intercolony contact, motile *B. subtilis* cells emerged from the mature biofilm to engulf the competing colony. Surfactin, Skf and Sdp then synergistically mediated the elimination of the competing *B. simplex* colony. The secretion of all relevant virulence factors was reinforced by a subpopulation of *B. subtilis* biofilm cells that lost their native pBS32 plasmid and exhibited increased aggressiveness against *B. simplex* cells. Similarly to *B. subtilis,* the fitness of naturally evolved genetic variants within *B. simplex* biofilms also changed during the interaction.

Importantly, the molecular mechanisms underlie the antagonistic interaction of *B. subtilis* and *B. simplex* turned out to be quite general. The synergistic and selective effect of the cannibalism toxins and surfactin towards competing *Bacillus* species, as well as a robust negative control of a native plasmid on the virulence of these effectors occurred under several conditions. It was evident on several biofilm growth media and during interspecies interaction between *B. subtilis* and the soilborne bacterium *Bacillus toyonensis*.

## Results

**Figure 1:**
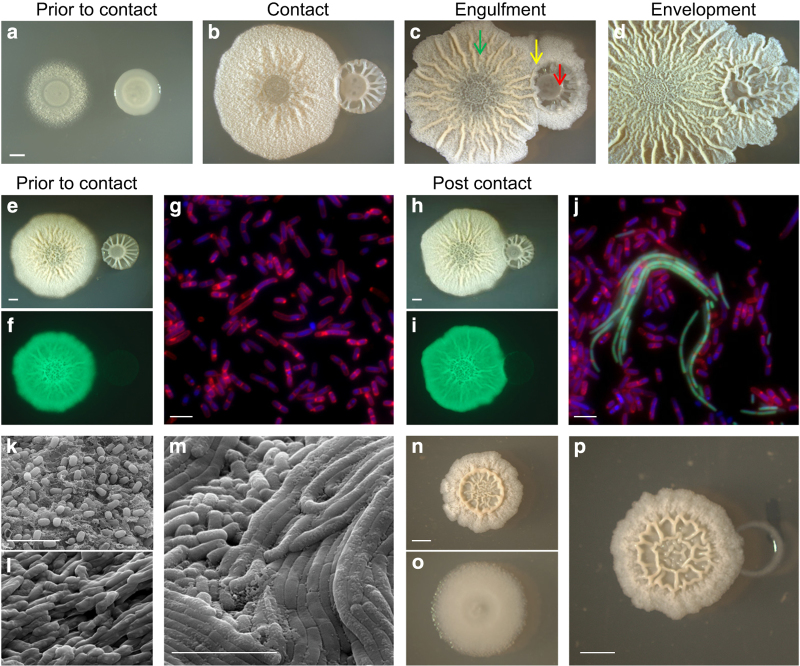
Antagonistic interaction between *B. subtilis* biofilms and competing *Bacillus* colonies. (**a**–**m**) Biofilms of *B. subtilis* and *B. simplex* grown at 30 °C on MSgg biofilm-inducing medium. (**a**–**d**) Biofilms of *B. simplex* were inoculated next to a *B. subtilis* biofilm at a distance of 0.8 cm. (**a**) Before contact (day 1), (**b**) contact (day 2), (**c**) engulfment (day 3) and (**d**) envelopment (day 4). Arrows indicate the different regions of the interaction: *B. subtilis* area (green), interphase (yellow) and *B. simplex* area (red), Scale bar represent 2 mm. (**e**–**j**) Biofilms of *B. simplex* were inoculated next to a biofilm of expressing GFP *B. subtilis* harbouring P_hyperspank_*-gfp* at a distance of 1 cm (before contact) and 0.8 cm (post contact), and grown for 2 days. (**e**,**h**) Bright field colonies images, scale bar represents 2 mm, (**f**,**i**) GFP fluorescence images of the colonies in **e** and **h**. (**g**,**j**) florescent microscope image: green—*B. subtilis* strain expressing GFP, red—membrane stain FM4–64, blue—4,6-diamidino-2-phenylindole DNA stain. Scale bar represents 5 μm. (**k**–**m**) environmental scanning electron microscopy images of *B. subtilis* and *B. simplex* biofilms grown for 3 days. Scale bars represent 5 μm. (**k**) *B. subtilis* grown separately. (**l**) *B. simplex* grown separately. (**m**) Interaction area of *B. subtilis* and *B. simplex* interacting biofilms in the engulfment stage. (**n**–**p**) *B. subtilis* and *B. toyonensis* colonies grown for 3 days at 30 °C on B4 biofilm medium. (**n**) *B. subtilis* grown separately. (**o**) *B. toyonensis* grown separately. (**p**) Interaction between *B. subtilis* and *B. toyonensis*, inoculated 0.3 cm apart. Scale bar represents 2 mm.

When grown on biofilm-inducing medium, both *B. subtilis* and *B. simplex* formed architecturally complex biofilms. *B. subtilis* created highly wrinkled complex biofilms, with a defined centre and dense ridges. *B. simplex,* isolated from soils, formed a wrinkled colony, with significant gaps between the thick wrinkles, and with a well-defined, smooth centre (Figure 1a–m and Supplementary Figure S1). When the two complex colonies came into proximity, and within a few hours of initial contact, a fragile ring of *B. subtilis* formed around *B. simplex* biofilm. The *B. subtilis* ring thickened with time, and engulfed the *B. simplex* biofilm (Figure 1a–d). Eventually, the entire surface of the engulfed colony was covered with *B. subtilis* matrix. To monitor this interaction at a single-cell resolution, the WT *B. subtilis* strain labelled with GFP was inoculated in proximity to *B. simplex* biofilms. Upon first contact, *B. subtilis* chains, significantly varying in length and number, invaded the *B. simplex* biofilm (Figure 1e–j). This variation might imply that *B. subtilis* cells are capable of replicating inside the *B. simplex* biofilm.

Using environmental scanning electron microscopy, the two biofilms could be clearly distinguished by their different extracellular matrix and the 3D organisation of their constituent cells (Figure 1k–m). The *B. subtilis* extracellular matrix was characterized by a fibrous web-like structure, while the *B. simplex* biofilm contained a thick extracellular matrix coating each cell in the biofilm. In addition, *B. simplex* biofilm cells were approximately twofold larger than *B. subtilis* biofilm cells. Upon direct contact, the *B. subtilis* and *B. simplex* interaction zone took on a different morphology than each isolated biofilm. This interaction zone was characterized by both an extremely dense extracellular matrix that heavily coated each of the interacting cells in the biofilm and by unique cell organisation patterns, and especially long cell chains (Figure 1m). Notably, *B. subitlis* cells form short-lived chains during biofilm growth, also producing the extracellular matrix.^35,36^ These cell chains disassemble within 48 h (Supplementary Figure 1 and ref. 35). As the scanning electron microscope revealed the presence of cell chains in 72 h biofilms, it is feasible that the invasion process into *B. simplex* induces chaining within aging *B. subitlis* biofilms.

We then asked whether *B. subtilis* biofilms can antagonise other *Bacillus* species. Thus, we studied the interaction between *B. subtilis* and the soil-bacterium *B. toyonensis*, highly related to *Bacillus cereus.* As *B. toyonensis* is not capable of growing on defined biofilm media, we chose to study the interaction on top of a rich solid biofilm media (B4).^37^ We found that within 3 days, *B. toyonensis* colony is mostly eradicated by *B. subtilis* (Figure 1n–p). These results led us to the conclusion that *B. subtilis* biofilms may be allopathic to competing *Bacillus* species. Thus, we decided to explore the interspecies interaction between *B. subtilis* colonies and competing colonies by evaluating the viability of the interacting partners.

**Figure 2:**
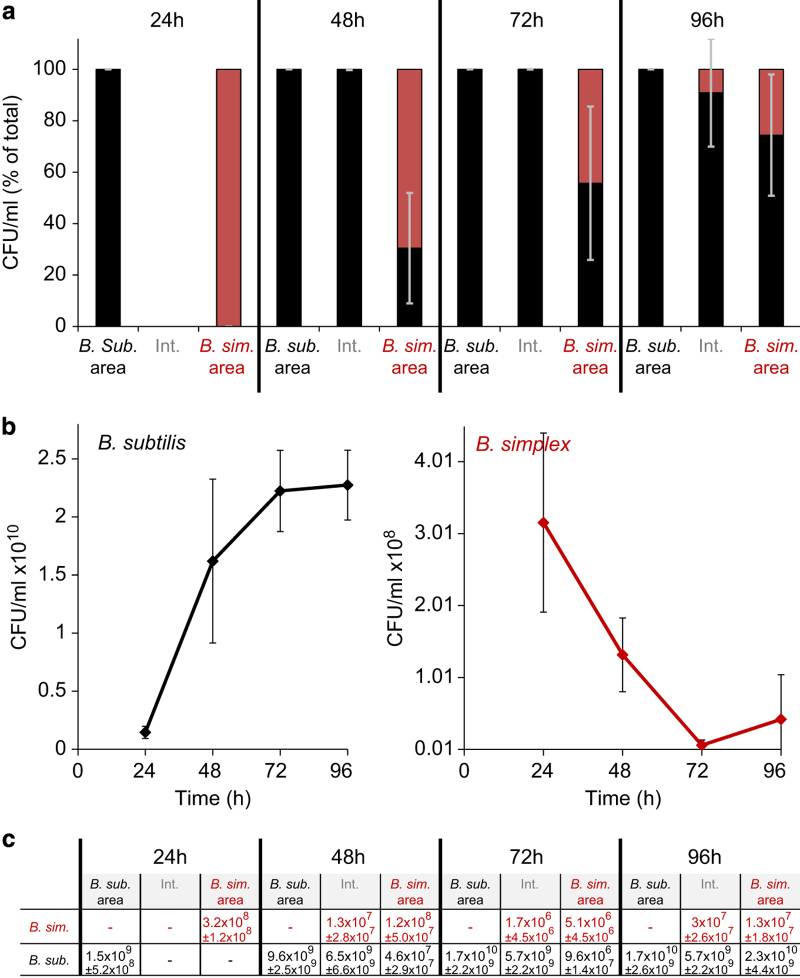
*B. subtilis* engulfment of and invasion into *B. simplex* biofilm mediates eradication of *B. simplex.* (**a**) Percentage of *B*. *subtilis* and *B. simplex* colony forming units at each stage of the interaction: the interacting colonies were divided into three sections: *B. sub.* area—*B. subtilis* section, Int.—interaction zone and *B. sim* area—*B. simplex* section. Each section was collected, sonicated and plated to determine the number of replicative cells of each species. In each section, percentage of each species from total cell number is presented (*n*=6). (**b**) Sum of the cell numbers from all sections as described in **a**, during 4 days of interaction (*n*=4). (**c**) Cell number from all sections as described in **a**, during 4 days of interaction (*n*=4). Error bars represent s.d.

Once the biofilms of *B. subtilis* and *B. simplex* contacted each other, a massive invasion of the *B. subtilis* cells into the *B. simplex* biofilm occurred, gradually leading to a nearly complete extinction of the *B. simplex* population (Figure 2). In contrast, when grown in isolation, the *B. simplex* population increased steadily (Supplementary Figure S2). Finally, after the envelopment stage, the absolute number of *B. subtilis* cells comprised over 90% of the total population within the former *B. simplex* colony area. Importantly, in the interaction zone, the number of multiplying viable *B. simplex* cells mildly increased, demonstrating that a subpopulation of *B. simplex* cells is resistant to *B. subtilis* killing (Figures 2b and c). This interaction occurred both on defined (Figures 1 and 2) and rich (Supplementary Figure S3) biofilm media.

**Figure 3:**
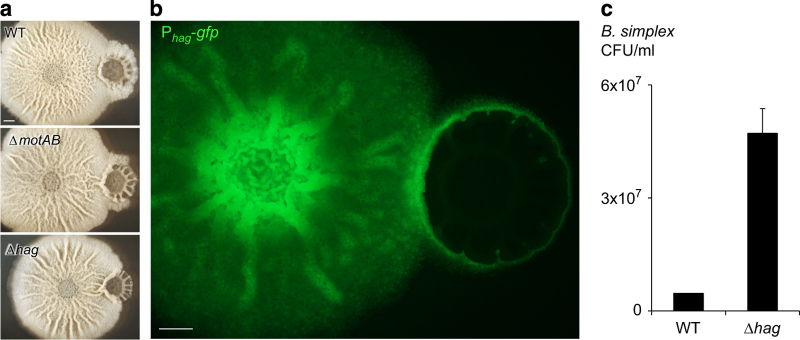
Expression of the motility genes is required for *B. subtilis* engulfment and killing of *B. simplex* biofilms. (**a**) upper—*B. subtilis* WT strain inoculated on MSgg biofilm-inducing medium at a distance of 0.8 cm from *B. simplex*. middle—*B. subtilis* Δ*motAB* mutant inoculated at distance of 0.8 cm from *B. simplex*. lower—*B. subtilis* Δ*hag* mutant inoculated at distance of 0.6 cm from *B. simplex*. Biofilms were grown for 3 days at 30 °C. Scale bars represent 2 mm. (**b**) P_hag_-*gfp* expression. *B. subtilis* P_*hag*_-*gfp* strain inoculated on biofilm-inducing medium at a distance of 0.8 cm from a *B.simplex* biofilm grown for 2 days at 30 °C. Scale bars represent 2 mm. (**c**) Colony forming units counts of *B. simplex* taken 3 days post inoculation next to *B. subtilis* WT (distance of 0.8 cm) or *B. subtilis* Δ*hag* mutant (distance of 0.6 cm), as in **a**. The interacting colonies were collected, sonicated and plated to determine the number of replicative cells. *n*=3, error bars represent s.d.

We then asked whether flagellated motility or flagella production have a role in the formation of the *B. subtilis* engulfment ring around *B. simplex.* Mature *B. subtilis* biofilms contain a small subpopulation of motile cells.^38^ When grown alone, deletion of the flagellin gene (Δ*hag*),^39^ yields somewhat smaller *B. subtilis* biofilms that are highly similar to the parental WT strain. In contrast, when we examined the ability of this non-motile mutant to engulf the *B. simplex* biofilms, the mutant froze in the engulfment ring stage (Figure 3). Characterisation of the motility expression, using a transcriptional fusion to the *hag* promoter, revealed that the *B. subtilis* cells that engulf *B. simplex* have an increased expression of motility, as judged by the expression of *hag* (Figure 3b and Supplementary Figure S4C). Furthermore, the ability of the non-motile Δ*hag* mutant to overcome and eliminate *B. simplex* was significantly reduced (Figure 3c). We then asked whether the motor unit proteins, MotA and MotB of the flagella are also important for engulfment of *B. simplex.* We found that a mutant in *motAB,* similarly to a flagellin mutant, had an apparent delay in engulfing the *B. simplex* colony. The delay was more modest than of a flagellin mutant, but highly reproducible (Figure 3a). These findings suggest that the spreading of *B. subtilis* structured colonies relies primarily on flagella production, while the rotation of the flagella has a secondary role when engulfing competing colonies.

Engulfment did not require a fully functional chemotaxis apparatus, as demonstrated using the Δ*cheA*, and Δ*cheY B. subtilis* mutants.^40^ Those mutants could successfully form an engulfment ring around *B. simplex*. The engulfment was also independent of swarming, as a mutant in *degU*, which is required for effective swarming,^41,42^ had little or no defect in the engulfment stage (Supplementary Figure S4A).

**Figure 4:**
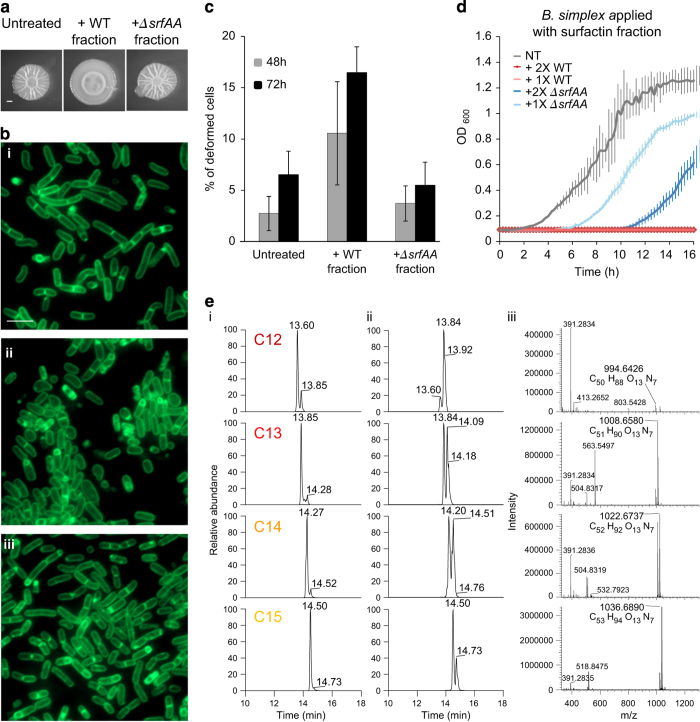
Surfactin mediates growth inhibition and cell deformation of *B. simplex*. (**a**) Biofilm formation of *B. simplex* grown for 3 days on biofilm-inducing plates, in the presence or absence of ×2 of *B. subtilis* supernatant fraction from either the WT or *srfAA* mutant, eluted in 100% methanol. Scale bars represent 2 mm. (**b**) Fluorescence microscope images of *B. simplex* biofilm cells grown for 3 days in the presence or absence of ×2 *B. subtilis* supernatant fraction from either the WT or *srfAA* mutant, eluted in 100% methanol, and stained with FM1–43. (i) Control, (ii) +WT fraction, (iii) +*srfAA* mutant fraction. Scale bar represents 2 μm. (**c**) Quantification of deformed *B. simplex* cells in *B. simplex* biofilms treated with or without ×2 *B. subtilis* supernatant fractions from WT or Δ*srfAA* mutant strains, eluted with 100% methanol. (*n*=5000). Error bars represent the s.d. (**d**) Growth curves of *B. simplex* in 96-well plates with shaking in MSgg. Cells were supplemented with supernatant fractions derived from either WT or *srfAA* mutant strains. (**e**) Liquid chromatography-mass spectrometry analysis of the varying tail length surfactin molecules found in the active surfactin fraction. (i) Chromatographs of surfactin standard (SIGMA S3523), (ii) chromatographs of the active surfactin fraction, (iii) mass spectrometry analysis of the varient surfactin molecules found in the active fraction.

The invasion of *B. subtilis* into *B. simplex* biofilms led to elimination of the cells within the invaded colony. As we found that the supernatants of post-logarithmic and stationary planktonic *B. subtilis* cultures led to complete inhibition of *B. simplex* growth (Supplementary Figure S5, Panel A), we attempted to purify bioactive *B. subtilis*-derived molecules that mediate the killing (for details, see Materials and Methods section). Importantly, the killing activity of the supernatant was density dependent, consistent with the idea that the killing factor(s) production can be regulated by quorum sensing, a phenomenon where bacterial behaviours are modulated in accordance with population density, through the synthesis and perception of small signalling molecules.^43^ When separating and purifying supernatant components on a gradient of organic solvent on a C-18 Sep-Pak cartridge as done previously for isolation of small bioactive compounds,^44,45^ we found that the most hydrophobic fraction, eluted in 100% methanol, had a strong inhibitory effect on *B. simplex* biofilm development, growth and biofilm-forming capacity (Figure 4a and Supplementary Figures S6A–C). The 80% methanol fraction showed a mild effect on *B. simplex* growth and development, while the rest of the fractions had no impact. When the cells treated with the bioactive 100% methanol fraction were examined under a florescence microscope, we found that the treated cells had severe morphological changes and abnormalities (Figure 4b). The cells were oval and deformed, in contrast to the elongated rod-shaped untreated cells. In addition, the cell membranes were severely damaged. We used liquid chromatography-mass spectrometry of the different fractions, and looked for known antibacterial compounds produced by *B. subtilis.* The liquid chromatography-mass spectrometry analysis demonstrated that the active 100% methanol fraction contained a high concentration of surfactin (Supplementary Figure S7). A lower concentration of surfactin was found in the slightly active 80% methanol fraction and only traces of surfactin were found in the non-active fraction. Unlike surfactin, the concentrations of iturin, fengycin,^46^ bacitracin,^47^ bacillibactin,^48^ plipastatin,^49^ subtilosin^50^ or bacillomycin F^51^ were not correlated with the killing activity. Consistent with our finding that the killing activity of the media is density dependent, surfactin production depends in the accumulation above a threshold of a peptide pheromone named ComX, accumulated late in the logarithmic stage.^15^ Indeed surfactin presence in the biofilm growth media was only evident after 8 h (Supplementary Figures S8 and 9).

The surfactin molecule with a C12 tail was only found in the active fractions, implying that the length of the tail affects the killing properties of the molecule. In order to verify the liquid chromatography-mass spectrometry results, supernatant from Δ*srfAA*, a *B. subtilis* mutant incapable of producing surfactin, were collected and fractionated. The 100% methanol fraction of the mutant supernatants had no effect on *B. simplex* growth and biofilm formation (Figure 4a,b). In addition, single cells treated with this fraction had no abnormalities in cell shape, in striking contrast to cells treated with the WT fraction (Figure 4a–c). In addition, the WT fraction fully inhibited *B. simplex* growth, while the equivalent fraction eluted from the Δ*srfAA* mutant only induced a moderate lag in the initiation of *B. simplex* growth (Figure 4d).

We then quantified the amount of surfactin in the supernatant. Using a commercially available standard, we showed that the purified fractions from *B. subtilis* are significantly more potent (Figure 4e and Supplementary Figure S10) than the commercially available surfactin. Consistent with our previous finding that short-tail isomers of surfactin are correlated with increased toxicity towards *B. simplex,* the ratio of short length (C12) to full length (C14–15) surfactin isomers was increased significantly in the purified surfactin fractions. Owing to the significantly increased potency of the surfactin purified from the conditioned media and the subtle effect on *B. simplex* growth observed upon exposure to high concentrations of the Δ*srfAA* mutant supernatant fractions, we speculated that additional bioactive killing molecules are secreted by *B. subtilis*.

**Figure 5:**
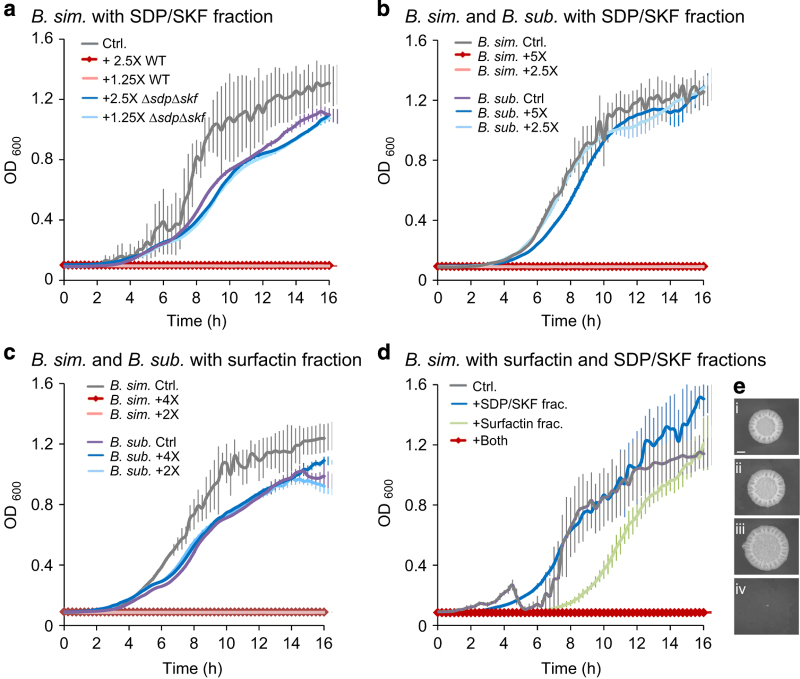
Surfactin and the cannibalism toxins inhibit *B. simplex* growth and biofilm formation at concentrations inert to *B. subtilis* itself and show synergistic cooperation. Growth curves of *B. simplex* or *B. subtilis* in liquid MSgg biofilm-inducing medium grown in 96-well plates, with shaking, at 30 °C. (**a**) Growth curves of *B. simplex*. Media supplemented with ×1.25 or ×2.5 *B. subtilis* supernatant protein fractions derived from WT or Δ*sdpC*Δ*skfA* mutant strain cultures, enriched using a 3 kDa Centricon. *n*=6 wells, error bars represent s.d. (**b**) Growth curves of *B. simplex* and *B. subtilis*. Media supplemented with ×2.5 or ×5 *B. subtilis* supernatant protein fractions, enriched using a 3 kDa centricon. *n*=6 wells, Error bars represent the s.d. (**c**) Growth curves of *B. simplex* and *B. subtilis*. Media supplemented with ×2 or ×4 *B. subtilis* supernatant surfactin fraction. *n*=6 wells, Error bars represent the s.d. (**d**) Growth curves of *B. simplex*. Media supplemented with: (i) sub-toxic concentrations of ×0.31 *B. subtilis* supernatant protein fractions, enriched using a 3 KDa Centricon, (ii) ×0.1 *B. subtilis* supernatant fraction eluted in 100% methanol, or (iii) a combination of both of these fractions. *n*=6 wells, Error bars represent the s.d. (**e**) Biofilm formation of *B. simplex* grown for 2 days on biofilm-inducing medium plates: (i) untreated, (ii) treated with ×0.625 *B. subtilis* active protein fraction, (iii) treated with ×0.5 *B. subtilis* supernatant fraction eluted with 100% methanol and (iv) treated with a combination of both fractions. Scale bar represents 2 mm.

The characterisation of these molecules was important to fully comprehend the attack mechanisms participating in this interspecies interaction. To this end, active proteins and/or peptides were eluted from the toxic supernatant using a Centricon Dialysis kit (Millipore). Concentrated supernatant products larger than 3 kDa had a strong inhibitory effect on *B. simplex* growth (Supplementary Figure S11). Mass spectrometry analysis of the protein fraction identified SdpC, a secreted toxin involved in bacterial cannibalism,^24^ as the active protein (Supplementary Figure S11B). An equivalent purified supernatant from a strain defective in production of both SdpC and SkfA (Δ*sdpC*Δ*skfA*), the two *B. subtilis* cannibalism toxins, had no effect on *B. simplex* growth (Figure 5a). Consistent with our finding that the killing activity of the supernatant of *B. subtilis* in a biofilm media was only significant after 8 h of growth, we found that the secretion of the cannibalism toxins was enhanced between 6 and 8 h of growth (Supplementary Figure S11C). Notably, a small residual activity of the protein fraction was evident after 6 h, consistent with our observation that the cannibalism toxins contribution to elimination of the competing *B. simplex* cells is more subtle that the contribution of surfactin.

We used a strain carrying the fusion of the *sdp* promoter with a luciferase gene to monitor the activity of this promoter in the presence and absence of *B. simplex*. Quite interestingly the expression of *sdpA-C*, encoding for SDP was found to be increased during the interaction with *B. simplex*. When the effect of a cell-free supernatant was compared with the effect of co-culturing, it became evident that co-culturing induces the expression of the *sdpA-C* promoter more strongly than the secretome of *B. simplex* (Supplementary Figure S12). These results can be consistent with our finding (Figure 2), that *B. simplex* colonies eradication is mediated by direct contact between the competing colonies.

To date, *B. subtilis* has mostly been thought to produce self-targeted cannibalism toxins, which act to enable survival and delay sporulation upon nutrient starvation. The cannibalism toxins were described previously as toxic for different bacterial species.^52,53^ However, a direct comparison of the potency of purified cannibalism toxins on *B. subtilis* and versus competing *Bacillus* species was the best of our knowledge never directly compared. To assess whether the *B. subtilis*-generated SdpC and SkfA cannibalism toxins and surfactin, induce a stronger effect against foreign *Bacillus* species than on *B. subtilis*, we grew *B. subtilis* and *B. simplex* in several concentrations of the SkfA and SdpC eluate (Figure 5b) and the surfactin fraction (Figure 5c). Both the surfactin fraction and the cannibalism toxins elute fully inhibited *B. simplex* growth at concentrations that failed to affect *B. subtilis*. It is still feasible that the cannibalism toxins can inhibit the growth of *B. subtilis* in much higher concentrations, when applied to a biofilm medium. In addition, surfactin, Skf and Sdp, had a dramatic synergistic effect on *B. simplex*; when added separately, low concentrations of the surfactin fraction and of the SKF-SDP eluate had only a mild effect on *B. simplex* growth (Figure 5d). However, when applied as a mixture they caused complete inhibition of *B. simplex* growth (Figure 5d). The surfactin fraction and the cannibalism toxins elute also synergistically inhibited biofilm development of *B. simplex* (Figure 5e).

Furthermore, mutants deleted for either *sdp*,*skf* operons or *srfAA* displayed a severely impaired attack process. These mutants partially took over the *B. simplex* biofilm, and showed a massive decrease in their ability to invade the *B. simplex* biofilm and a subtle defect in overcoming *B. simplex*. However a triple mutant for *srfAA*, *skf* and *sdp* had the most substantial defect in killing *B. simplex* consistent with the idea that SKF, SDP and surfactin have synergistic killing activity during the interaction (Supplementary Figure S13).

We then asked whether surfactin, SDP and SKF were also at the core of the antagonistic interaction with additional *Bacillus* species. When we examined the effect of the *B. subtilis* supernatant on the bacterium *B. toyonensis* we found it is extremely toxic, when collected from the WT, and lacks killing activity when purified from a triple mutant for the production of surfactin, SDP and SKF (Supplementary Figure S14C). These results imply that similar mechanisms enable the allopathic interaction of *B. subtilis* biofilms with *B*. *toyonensis* (Figure 1n–p). These killing mechanisms were also used by *B. subtilis* when we tested the interaction with *B. simplex* on top of a rich solid biofilm media (B4) (Supplementary Figures S14A and B).

**Figure 6:**
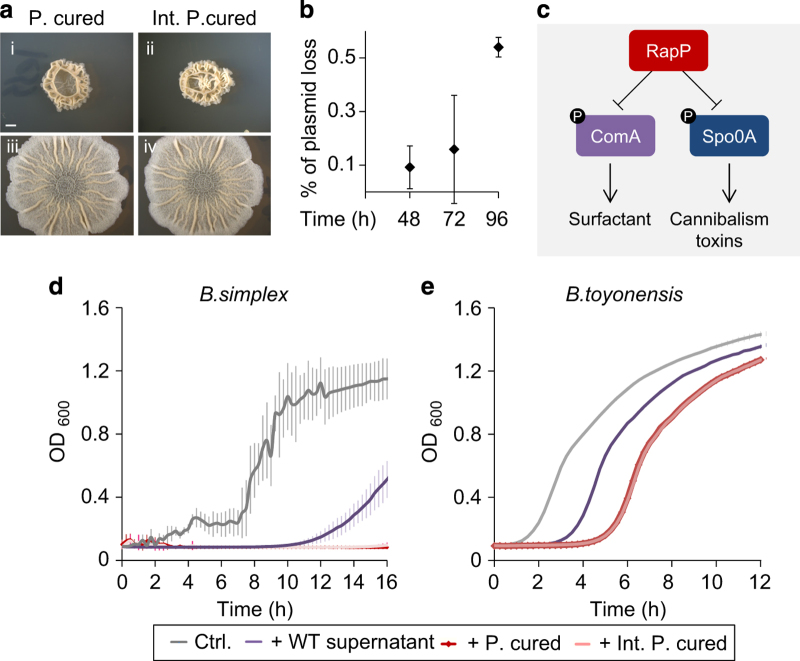
Loss of the natural *B. subtilis* plasmid increases the production of *B. subtilis* killing factors that inhibit rival *Bacillus* species growth (**a**) (i) *B. subtilis* plasmid-cured strain. (ii) *B. subtilis* plasmid-cured strains isolated from interacting *B. subtilis-B. simplex* biofilms. (iii, iv) Genetic Complimentation with the *rapP* for the strains in (i) and (ii) respectively. (**b**) Percentage of *B. subtilis* biofilms cells that lost their natural plasmid during the interaction with *B. simplex*. *n*=6, error bars represent s.d. (**c**) The role of RapP in regulation of the killing factors that inhibit rival *Bacillus* species growth. (**d**, **e**) Growth curves of *Bacillus* species exposed to supernatant of *B. subtilis* WT, plasmid-cured, and interaction evolved strain. Bacteria were grown in 96-well plates, with shaking, at 30 °C. (**b**) *B. simplex* growth curves. MSgg medium supplemented with 8-h ×0.25 *B. subtilis* supernatants. *n*=6, error bars represent s.d. (**d**) Growth curves of *B. toyonensis*. Liquid LB broth medium, supplemented with 8-h ×0.25 *B. subtilis* supernatants. *n*=6, error bars represent the s.d.

Strikingly, in the interaction with *B. simplex* we found an increasing occurrence of spontaneous mutations in the *B. subtilis* biofilm. Significantly more mutants were formed in interacting colonies in the final stages of the interaction compared with the initial stages (Figure 6b and Supplementary Figure S15A). In addition, all *B. subtilis* mutants were characterized by hyperrugose biofilms, reminiscent of biofilms formed by *B. subtilis* strains cured from their natural plasmid (Figure 6a). A whole-genome sequencing analysis showed that all the hyperrugose biofilm mutants did not carry any mutation in their genome, indicating that the hyperrugose phenotype may be an outcome of *B. subtilis* plasmid loss. A PCR analysis demonstrated that the evolved strains lacked *rapP*, a phosphatase whose encoding gene is carried on the plasmid pBS32 (Supplementary Figure S15B), and dephosphorylates Spo0F, resulting in activation of extracellular matrix production (Figure 6c). As we suspected that the hyperrugose phenotype is due to loss of RapP, we complemented the mutants for the *rapP* gene (Figure 6a), which led to full restoration of the biofilm morphology.

The supernatants of plasmid-cured mutants demonstrated increased killing capacity toward *B. simplex*, when compared with those of the WT parental strain. The same phenotype was observed in a WT strain was artificially cured from the plasmid (Figure 6d). Strikingly, the plasmid loss also increased the toxicity of *B. subtilis* supernatant towards *B. toyonensis* (Figure 6e).

Importantly, an increase in Spo0F and ComA phosphorylation were previously observed in the plasmid-cured strain,^33,54^ thus indicating that a significant increase in the levels of Surfactin, SKF and SDP also elevates the fitness of plasmid-cured strains during interspecies competition.

Importantly, interspecies competition also affected the rate and the nature of the acquired mutated phenotypes in *B. simplex* biofilms. When *B. simplex* is grown alone, mutants can be isolated from mature biofilms, which show different levels of biofilm defects (Supplementary Figure S16). Quite strikingly, during the interspecies interaction, only mutants in which biofilm formation was fully inhibited were detected, suggesting that their competitive advantage was greater than those forming partially defective biofilms (Identified mutations are demonstrated in Supplementary Figure S16). Many of the biofilm inhibited *B. simplex* mutants enriched during interspecies interactions were associated with the loss of Spo0A (Supplementary Figure S16).

## Discussion

Soil bacteria have developed diverse mechanisms to ensure their survival in harsh competitive soil environments. Some activities, such as generation and secretion of small active secondary metabolites, and the development of structurally complex colonies, present new therapeutic targets. Understanding interspecies interactions is also an important tool in comprehending bacterial developmental programs.^8,55^ The bacterial physiology is greatly affected by its surrounding environment, where the interaction between species can modify bacterial gene expression patterns and induce the secretion of various antimicrobial molecules.^55,56^

We chose to study the interaction between *B. subtilis* and *B. simplex*, two soil bacteria that form structured communities. These closely related bacteria can generate three dimensional complex biofilm structures while competing for the same ecological niche. Competition between the two species leads to *B. subtilis* engulfment and elimination of the *B. simplex* colony and enables *B. subtilis* to take over the ecological niche. The contact between the two biofilms leads to changes in the morphology and composition of the cells in the interaction zone, suggesting interspecies signalling.

Importantly, engulfment requires functional flagella, a finding that is consistent with the abundance of motile cells within mature *B. subtilis* biofilms (up to 10%, (ref. 26) data not shown). Although a non-motile mutant in the flagellin protein had little or no biofilm defect when grown in isolation, it was incapable of engulfing and overcoming neighbouring *B. simplex* colony. These finding imply that a reservoir of flagellated cells is actively maintained in *B. subtilis* biofilms, overcoming the negative-feedback loops downregulating motility in the single-cell level, and improving the fitness of the biofilm population during interspecies competition. Interestingly, the accumulation of motile cells in the interaction area was correlated with the formation of a thick wrinkle in the interphase (Figure 1d,m and Figure 3b). It was suggested previously that channels exist within *B. subtilis* wrinkles,^57^ and facilitate the transport of liquids^57^ and of motile cells.^58^ Thus, it is feasible that the transport of *B. subtilis* bioactive killing factors into competing *Bacillus* colonies and cross-species interactions may be enhanced by localised formation of channels.

Three secreted factors were shown to have a central role in elimination of competing *Bacillus* colonies: the small biosurfactant surfactin, the SdpC protein and the SkfA peptide. All three are considered autoinducers, affecting the behaviour of *B. subtilis* itself. It has been previously shown that *B. subtilis* changes its membrane phospholipid composition during secretion of surfactin, in order to defend itself against the membrane disruption activity of surfactin.^59^ Our results imply that the C-12-long surfactin carbon tail is correlated with the strongest effect on *B. simplex* cells. The length of the surfactin tail may act differently on different bacterial species, and surfactin synthesis patterns may be dictated by the specific bacterial species interacting with *B*. *subtilis*.

Both the SdpC protein and SkfA peptide are autoinducers that allow a subpopulation of *B. subtilis* cells expressing the master-regulator Spo0A, to eliminate those *B*. *subtilis* cells that did not commit to Spo0A-dependent programs. Thus, the primary role of these factors has been associated with postponement of sporulation during nutrient starvation.^60^ Alternatively, cannibal cells were suggested to eliminate ‘cheater’ cells not expressing extracellular matrix components during biofilm formation in mutants defective in cell-wall modulation, but had no phenotype in a WT background.^25^ We found that these molecules are orders of magnitude more potent towards competing *Bacillus* species, such as *B. simplex*, than to *B. subtilis* itself. Potency towards planktonically growing *B. subtilis* cells, biofilm state *B. subtilis*, and stationary *B. subtilis* cells remained relatively low. Similar results were observed with surfactin. Thus, it is highly feasible that the primary role of SdpC, SkfA and surfactin is to act against rival colonies in the soil. We suggest that the various adaptations of *B. subtilis*, such as the ability to both change its membrane characteristics to gain surfactin resistance, and to generate immunity factors against the cannibalism toxins, provide *B*. *subtilis* a unique ability to use autoinducers dually—as an weapon as well as self-regulatory mechanism. Importantly, we found that these three active factors are much more potent when working together, which may explain why they are all temporally co-expressed during biofilm maturation. The synergistic killing properties of these effectors may be mediated by the pore-forming surfactin, which facilitates its penetration into the rival biofilm extracellular matrix. This penetration can enable the diffusion of SdpC and SkfA into various layers of the rival biofilm that then mediate bacterial killing in these areas. In addition, it is feasible that surfactin promotes the entry of the polar SKF and SDP peptides into rival biofilm cells.

In numerous studies, plasmid acquisition was shown to be an effective means of gaining virulence properties, improving fitness during an interspecies competition.^61,62^ Quite strikingly, a reverse strategy is used by *B. subtilis* when encountering competing *Bacillus* species. During interspecies interaction, *B*. *subtilis* showed increased loss of its natural plasmid, which led to increased secretion of virulence factors SdpC, SkfA and surfactin. The increased secretion is due to the loss of the phosphatase, RapP, encoded on the plasmid, and subsequent increase in the phosphorylation of the master regulators ComA, that activates genetic competence and surfactin secretion, and Spo0A, that regulates the production of SKF and SDP.^33,54,63^ As the loss only occurs in a subpopulation of the biofilm cell community, it is highly feasible that plasmid loss is a strategy to temporary increase the overall fitness of the multicellular community during interspecies interactions, until the plasmid is re-gained following alleviation of the selective pressure. The mechanisms underlying plasmid loss and its regulation remain to be determined.

Overall, our results demonstrate how interspecies interactions contribute to the shaping of the expression patterns and genetic organisation of rival bacterial species. More specifically, we show that *B. subtilis* is much more dependent on its motile cells subpopulation, autoinducers, and on its capacity to lose a plasmid-encoded master regulator, in order to overcome a rival biofilm during an interspecies interaction, compared with the dependence on the same factors when grown in isolation.

## Materials and Methods

### Strains and media

All experiments were performed with *B. subtilis* NCIB 3610 (ref. 64) strain, *B. simplex* WT strain and their derivatives. For cloning purposes, we used *B. subtilis* strain PY79 and *Escherichia* *coli* strain DH5α. A complete list is shown in Supplementary Table S1.

Strains and plasmids were constructed using standard methods.^64,65^ Oligonucleotides used for PCR in this study are listed in Supplementary Table S2. All deletion mutations were generated by long-flanking homology PCR mutagenesis.^66^ DNA was first introduced by transformation into strain PY79 and the deletion further integrated into NCIB 3610 by transformation as described previously.^67^

The strains were routinely manipulated in LB broth (Difco, Le Pont de Claix, France), B4 was prepared as described previously,^37^ mLB solid medium (modified LB)^68^ or MSgg medium (5 m mol/l potassium phosphate, 100 m mol/l MOPS pH 7, 2 m mol/l MgCl_2_, 50 μ mol/l MnCl_2_, 125  μ mol/l FeCl_3_, 700  μ mol/l CaCl_2_, 1  μ mol/l ZnCl_2_, 2  μ mol/l thiamine, 0.5% glycerol, 0.5% glutamate, 50 μg/ml threonine, tryptophan and phenylalanine). Solid medium contained 1.5% bacto agar (Difco). Note that the iron concentration in the solid MSgg medium was 2.5-fold higher compared with the original recipe.^64^ Selective media were prepared as described in Supplementary Materials and Methods.

### Interaction assay

For details, please refer to the Supplementary Text and Supplementary Materials and Methods.

### Quantification of plasmid-cured cells

To analyse the percentage of cells that lost their natural plasmid during the interaction between *B. subtilis* and *B. simplex*, interaction plates were incubated for the required time period. The biofilms were collected, inserted into 200 μl phosphate-buffered saline and mildly sonicated. The cells were then diluted, plated and incubated at 37 °C overnight to allow the formation of colonies. Colonies were then further incubated at 23 °C overnight. The plasmid-cured colonies were recognised by their colony morphology.^32^ The suspected colonies were confirmed on MSgg-agar plates. PCR analysis confirmed that the evolved colonies lacked the *rapP* gene.

### Supernatant production

For details, please refer to the Supplementary Text and Supplementary Materials and Methods.

### Separation and identification of surfactin

The growth inhibiting, 100% methanol elute of the 8-h-old *B. subtilis* conditioned medium was eluted from a C-18 SPE column (Waters, Milford, MA, USA). A single *B. subtilis* WT colony, isolated on a solid LB plate, was inoculated into 3 ml of LB broth, grown overnight at room temperature. The overnight culture (100 μl) were inoculated into 100 ml MSgg and grown in a 300 l flask at 37 °C with shaking for 8 h. The conditioned medium was centrifuged at 8,000 r.p.m. for 10 min; supernatant was removed and filtered through a 0.22-μm filter. For further purification the supernatant was fractionated on a C-18 Sep-Pak cartridge using stepwise elution of 0–100% methanol with steps of 10%–20%–30%–40%–60%–80%–100%. The methanol fractions were evaporated by speed-vac. To test the activity of the extracts, the evaporated samples were dehydrated with dW to a final concentration of 20×. The active fraction was further analysed using liquid chromatography-mass spectrometry as described in Supplementary Materials and Methods.

### Separation of SdpC and SkfA peptides

Protein fraction of the conditioned medium from 8-h-old *B. subtilis* was fractionised by Cellulose Amicon Ultra Centrifugal Filter Unit with Ultracel-3 Membrane, 15 ml Capacity, 3 kDa nominal molecular weight limit (NMWL; Millipore, Cork, Ireland). For details please refer to Supplementary Materials and Methods.

### Growth measurements

A single colony of *B. subtilis* or *B. simplex*, isolated on a solid LB plate, was inoculated into 3 ml of LB broth and grown to a mid-logarithmic phase of growth (*B. subtilis* 4 h, *B. simplex* 5 h at 37 °C with shaking). Cells were diluted 1:100 in 150 μl liquid MSgg medium of each well of a 96-well microplate (Thermo Scientific, Roskilde, Denmark). Cells were grown with agitation at 30 °C for 16 h in a microplate reader (Synergy 2, BioTek, Winooski, VT, USA), and the optical density at 600 nm (OD_600_) was measured every 15 min.

### Assessing the effects of *B. subtilis* bioactive effectors on biofilm development and cell growth

For details, please refer to the Supplementary Text and Supplementary Materials and Methods.

### Fluorescence microscopy

To analyse the effect of contact between the two biofilms, *B. subtilis* WT or its indicated derivatives and *B. simplex*, interaction plates were prepared as described, and incubated for the required time period. The interacting biofilms were divided to three areas as mentioned in the analysis of bacterial population from the interaction. The *B. simplex* area was separated from the plate and suspended in 200 μl phosphate-buffered saline. The *B. simplex* biofilm was roughly disassembled by pipetting. Samples were centrifuged briefly, and re-suspended in 10 μl of 1× phosphate-buffered saline supplemented with the membrane stain FM4–64 (Molecular Probes, Eugene, OR, USA) at 1 μg/ml and the DNA stain 4,6-diamidino-2-phenylindole (Sigma, Saint-Louis, MO, USA) at 2 μg/ml. This concentrated cell suspension (3 μl) was placed on a microscope slide. A freshly prepared poly l-lysine-treated (Sigma) coverslip. Treated coverslip was used to monitor the *B. subtilis* GFP marked strain invasion to the *B. simplex* biofilm area. The cells were observed by Axio microscope (Zeiss, Goettingen, Germany) images were analysed by Zen-10 software (Zeiss).

To analyse the effect of the bioactive fraction eluted with methanol on *B. simplex* biofilms in the single-cell level, the biofilm was roughly disassembled by pipetting. Samples were centrifuged briefly, and re-suspended in 10 μl of 1× phosphate-buffered saline supplemented with the membrane stain FM1–43 (Molecular Probes) at 1 μg/ml and the DNA stain 4,6-diamidino-2-phenylindole (Sigma) at 2 μg/ml. Importantly. This concentrated cell suspension (3 μl) was placed on a microscope slide. A freshly prepared poly l-lysine-treated (Sigma) coverslip was used to immobilise the cells for membrane visualisation. The cells were observed by Axio microscope (Zeiss) images were analysed by Zen-10 software.

### Environmental scanning electron microscopy

Biofilms grown for 72 h at 30 °C were fixed overnight at 4 °C with 2% glutaraldehyde, 3% papaformaldehyde, 0.1 mol/l Sodium Cacodylate (pH 7.4), 5 m mol/l CaCl_2_. After two 15 min washes with double distilled water, samples were dehydrated through series of ethanol washes. Subsequently, samples were dried on filter paper (Whatman) overnight at room temperature, mounted and stored under vacuum. Mounted samples were sputter coated with gold–palladium shortly before examination with a scanning electron microscope ×L30 with field emission gun.

### Estimating the effect of interspecies interaction on bacterial evolution

Interacted biofilms, treated as described in analysis of bacterial populations in the interaction, were divided, diluted and plated on LB-agar plates. The bacterial colonies from the LB-agar plates were inoculated into 3 ml of LB broth and grown to a mid-logarithmic phase of growth. A measure of 2 μl of each culture was inoculated onto MSgg-agar plates and incubated at 30 °C for 48 h. The bacterial biofilm were screened for biofilm defects. Biofilms with hereditable morphological defects upon re-inoculation were characterized as biofilm mutants. The mutants generated as a result of biofilms interactions were compared with mutants from of *B. subtilis* and *B. simplex* biofilms that were grown in isolation.

### *RapP* complementation

For details, please refer to the Supplementary Text and Supplementary Materials and Methods.

### Alignment of *B. subtilis* reads and identification of mutations

For details, please refer to the Supplementary Text and Supplementary Materials and Methods.

### *B. simplex* genome assembly and mutation identification

For details, please refer to the Supplementary Text and Supplementary Materials and Methods.

## References

[bib1] Tringe, S. G. et al. Comparative metagenomics of microbial communities. Science 308, 554–557 (2005). 15845853 10.1126/science.1107851

[bib2] Torsvik, V. , Goksøyr, J. & Daae, F. L. High diversity in DNA of soil bacteria. Appl. Environ. Microbiol. 56, 782–787 (1990). 2317046 10.1128/aem.56.3.782-787.1990 PMC183421

[bib3] Wardle, D. A. et al. Ecological linkages between aboveground and belowground biota. Science 304, 1629–1633 (2004). 15192218 10.1126/science.1094875

[bib4] Lynch, J. M. et al. Microbial diversity in soil: ecological theories, the contribution of molecular techniques and the impact of transgenic plants and transgenic microorganisms. Biol. Fertil. Soils 40, 363–385 (2004).

[bib5] Kolter R. & Greenberg E. P. Microbial sciences: the superficial life of microbes. Nature 441, 300–302 (2006). 16710410 10.1038/441300a

[bib6] Elias, S. & Banin, E. Multi-species biofilms: living with friendly neighbors. FEMS Microbiol. Rev. 36, 990–1004 (2012). 22229800 10.1111/j.1574-6976.2012.00325.x

[bib7] Little, A. E. F. , Robinson, C. J. , Peterson, S. B. , Raffa, K. F. & Handelsman, J. Rules of engagement: interspecies interactions that regulate microbial communities. Ann. Rev. Microbiol. 62, 375–401 (2008). 18544040 10.1146/annurev.micro.030608.101423

[bib8] Vlamakis, H. , Chai, Y. , Beauregard, P. , Losick, R. & Kolter, R. Sticking together: building a biofilm the Bacillus subtilis way. Nat. Rev. Microbiol. 11, 157–168 (2013). 23353768 10.1038/nrmicro2960 PMC3936787

[bib9] Beloin, C. , Renard, S. , Ghigo, J.-M. & Lebeaux, D. Novel approaches to combat bacterial biofilms. Curr. Opin. Pharmacol. 18, 61–68 (2014). 25254624 10.1016/j.coph.2014.09.005

[bib10] Earl, A. M. , Losick, R. & Kolter, R. Ecology and genomics of Bacillus subtilis. Trends Microbiol. 16, 269–275 (2008). 18467096 10.1016/j.tim.2008.03.004 PMC2819312

[bib11] Sikorski, J. & Nevo, E. Patterns of thermal adaptation of Bacillus simplex to the microclimatically contrasting slopes of 'Evolution Canyons' I and II, Israel. Environ. Microbiol. 9, 716–726 (2007). 17298371 10.1111/j.1462-2920.2006.01193.x

[bib12] Dubnau, D. Genetic competence in Bacillus subtilis. Microbiol. Rev. 55, 395–424 (1991). 1943994 10.1128/mr.55.3.395-424.1991 PMC372826

[bib13] Dubnau, D. & Provvedi, R. Internalizing DNA. Res. Microbiol. 151, 475–480 (2000). 10961462 10.1016/s0923-2508(00)00166-2

[bib14] Roggiani, M. & Dubnau, D. ComA, a phosphorylated response regulator protein of Bacillus subtilis, binds to the promoter region of srfA. J. Bacteriol. 175, 3182–3187 (1993). 8387999 10.1128/jb.175.10.3182-3187.1993 PMC204641

[bib15] Magnuson, R. , Solomon, J. & Grossman, A. D. Biochemical and genetic characterization of a competence pheromone from B. subtilis. Cell 77, 207–216 (1994). 8168130 10.1016/0092-8674(94)90313-1

[bib16] Gonzalez, D. J. et al. Microbial competition between Bacillus subtilis and Staphylococcus aureus monitored by imaging mass spectrometry. Microbiology 157, 2485–2492 (2011). 21719540 10.1099/mic.0.048736-0 PMC3352172

[bib17] Falardeau, J. , Wise, C. , Novitsky, L. & Avis, T. J. Ecological and mechanistic insights into the direct and indirect antimicrobial properties of Bacillus subtilis lipopeptides on plant pathogens. J. Chem. Ecol. 39, 869–878 (2013). 23888387 10.1007/s10886-013-0319-7

[bib18] Avigad, L. S. & Bernheimer, A. W. Nature and properties of a cytolytic agent produced by Bacillus subtilis. J. Gen Microbiol. 61, 361–369 (1970). 4992273 10.1099/00221287-61-3-361

[bib19] Hoefler B. C. , Gorzelnik K. V. , Yang J. Y. , Hendricks N. & Dorrestein P. C . Enzymatic resistance to the lipopeptide surfactin as identified through imaging mass spectrometry of bacterial competition. Proc. Natl Acad. Sci. USA 109, 13082–13087 (2012). 22826229 10.1073/pnas.1205586109 PMC3420176

[bib20] Watrous, J. et al. Mass spectral molecular networking of living microbial colonies. Proc. Natl Acad. Sci. USA 109, E1743–E1752 (2012). 22586093 10.1073/pnas.1203689109 PMC3387089

[bib21] Grau, A. , Go, J. C. & Ortiz, A. A study on the interactions of surfactin with phospholipid vesicles. Biochim. Biophys. Acta 1418, 307–319 (1999). 10320682 10.1016/s0005-2736(99)00039-5

[bib22] Chai, Y. , Chu, F. , Kolter, R. & Losick, R. Bistability and biofilm formation in Bacillus subtilis. Mol. Microbiol. 67, 254–263 (2008). 18047568 10.1111/j.1365-2958.2007.06040.x PMC2430929

[bib23] Lopez, D. , Fischbach, M. A. , Chu, F. , Losick, R. & Kolter, R. Structurally diverse natural products that cause potassium leakage trigger multicellularity in Bacillus subtilis. Proc. Natl Acad. Sci. USA 106, 280–285 (2009). 19114652 10.1073/pnas.0810940106 PMC2629187

[bib24] Ellermeier, C. D. , Hobbs, E. C. , Gonzalez-Pastor, J. E. & Losick, R. A three-protein signaling pathway governing immunity to a bacterial cannibalism toxin. Cell 124, 549–559 (2006). 16469701 10.1016/j.cell.2005.11.041

[bib25] Lopez, D. , Vlamakis, H. , Losick, R. & Kolter, R. Cannibalism enhances biofilm development in Bacillus subtilis. Mol. Microbiol. 74, 609–618 (2009). 19775247 10.1111/j.1365-2958.2009.06882.x PMC2983100

[bib26] Vlamakis, H. , Aguilar, C. , Losick, R. & Kolter, R. Control of cell fate by the formation of an architecturally complex bacterial community. Genes Dev. 22, 945–953 (2008). 18381896 10.1101/gad.1645008 PMC2279205

[bib27] Branda, S. S. , Chu, F. , Kearns, D. B. , Losick, R. & Kolter, R. A major protein component of the Bacillus subtilis biofilm matrix. Mol. Microbiol. 59, 1229–1238 (2006). 16430696 10.1111/j.1365-2958.2005.05020.x

[bib28] Chu, F. , Kearns, D. B. , Branda, S. S. , Kolter, R. & Losick, R. Targets of the master regulator of biofilm formation in Bacillus subtilis. Mol. Microbiol. 59, 1216–1228 (2006). 16430695 10.1111/j.1365-2958.2005.05019.x

[bib29] Hobley, L. et al. BslA is a self-assembling bacterial hydrophobin that coats the Bacillus subtilis biofilm. Proc. Natl Acad. Sci. USA 110, 13600–13605 (2013). 23904481 10.1073/pnas.1306390110 PMC3746881

[bib30] Kearns, D. B. , Chu, F. , Branda, S. S. , Kolter, R. & Losick, R. A master regulator for biofilm formation by Bacillus subtilis. Mol. Microbiol. 55, 739–749 (2005). 15661000 10.1111/j.1365-2958.2004.04440.x

[bib31] Bai, U. , Mandic-Mulec, I. & Smith, I. SinI modulates the activity of SinR, a developmental switch protein of Bacillus subtilis, by protein-protein interaction. Genes Dev. 7, 139–148 (1993). 8422983 10.1101/gad.7.1.139

[bib32] McLoon, A. L. , Guttenplan, S. B. , Kearns, D. B. , Kolter, R. & Losick, R. Tracing the domestication of a biofilm-forming bacterium. J. Bacteriol. 193, 2027–2034 (2011). 21278284 10.1128/JB.01542-10 PMC3133032

[bib33] Parashar, V. , Konkol, M. A. , Kearns, D. B. & Neiditch, M. B. A plasmid-encoded phosphatase regulates Bacillus subtilis biofilm architecture, sporulation, and genetic competence. J. Bacteriol. 195, 2437–2448 (2013). 23524609 10.1128/JB.02030-12 PMC3650544

[bib34] Omer Bendori, S. , Pollak, S. , Hizi, D. & Eldar, A. The RapP-PhrP quorum-sensing system of Bacillus subtilis strain NCIB3610 affects biofilm formation through multiple targets, due to an atypical signal-insensitive allele of RapP. J. Bacteriol. 197, 592–602 (2015). 25422306 10.1128/JB.02382-14 PMC4285980

[bib35] Chai, Y. , Kolter, R. & Losick, R. Reversal of an epigenetic switch governing cell chaining in Bacillus subtilis by protein instability. Mol. Microbiol. 78, 218–229 (2010). 20923420 10.1111/j.1365-2958.2010.07335.x PMC2998389

[bib36] Norman, T. M. , Lord, N. D. , Paulsson, J. & Losick, R. Memory and modularity in cell-fate decision making. Nature 503, 481–486 (2013). 24256735 10.1038/nature12804 PMC4019345

[bib37] Barabesi, C. et al. Bacillus subtilis gene cluster involved in calcium carbonate biomineralization. J. Bacteriol. 189, 228–235 (2007). 17085570 10.1128/JB.01450-06 PMC1797216

[bib38] Houry, A. et al. Bacterial swimmers that infiltrate and take over the biofilm matrix. Proc. Natl Acad. Sci. USA 109, 13088–13093 (2012). 22773813 10.1073/pnas.1200791109 PMC3420162

[bib39] Lavallie, E. R. & Stahl, M. L. Cloning of the flagellin gene from bacillus subtilis and complementation studies of an in vitro-derived deletion mutation. J. Bacteriol. 171, 3085–3094 (1989). 2498283 10.1128/jb.171.6.3085-3094.1989 PMC210019

[bib40] Rao, C. V. , Glekas, G. D. & Ordal, G. W. The three adaptation systems of Bacillus subtilis chemotaxis. Trends. Microbiol. 16, 480–487 (2008). 18774298 10.1016/j.tim.2008.07.003 PMC3532902

[bib41] Kearns, D. B. , Chu, F. , Rudner, R. & Losick, R. Genes governing swarming in Bacillus subtilis and evidence for a phase variation mechanism controlling surface motility. Mol. Microbiol. 52, 357–369 (2004). 15066026 10.1111/j.1365-2958.2004.03996.x

[bib42] Verhamme, D. T. , Kiley, T. B. & Stanley-Wall, N. R. DegU co-ordinates multicellular behaviour exhibited by Bacillus subtilis. Mol. Microbiol. 65, 554–568 (2007). 17590234 10.1111/j.1365-2958.2007.05810.x

[bib43] Ng, W. L. , Bassler, B. L. Bacterial quorum-sensing network architectures. Annual Rev. Genet. 43, 197–222 (2009). 19686078 10.1146/annurev-genet-102108-134304 PMC4313539

[bib44] Kolodkin-Gal, I. , Hazan, R. , Gaathon, A. , Carmeli, S. & Engelberg-Kulka, H. A linear pentapeptide is a quorum-sensing factor required for mazEF-mediated cell death in Escherichia coli. Science 318, 652–655 (2007). 17962566 10.1126/science.1147248

[bib45] Kumar, S. , Kolodkin-Gal, I. & Engelberg-Kulka, H. Novel quorum-sensing peptides mediating interspecies bacterial cell death. mBio 4, e00314–00313 (2013). 23736285 10.1128/mBio.00314-13 PMC3668371

[bib46] Kim P. I. , Ryu J. , Kim Y. H. & Chi Y. T. Production of biosurfactant lipopeptides iturin A, fengycin, and surfactin A from Bacillus subtilis CMB32 for control of colletotrichum gloeosporioides. J. Microbiol. Biotechnol. 20, 138–145 (2010). 20134245

[bib47] Ming, L. J. & Epperson, J. D. Metal binding and structure-activity relationship of the metalloantibiotic peptide bacitracin. J. Inorg. Biochem. 91, 46–58 (2002). 12121761 10.1016/s0162-0134(02)00464-6

[bib48] May, J. J. , Wendrich, T. M. & Marahiel, M. A. The dhb operon of Bacillus subtilis encodes the biosynthetic template for the catecholic siderophore 2,3-dihydroxybenzoate-glycine-threonine trimeric ester bacillibactin. J. Biol. Chem. 276, 7209–7217 (2001). 11112781 10.1074/jbc.M009140200

[bib49] Volpon, L. , Besson, F. & Lancelin, J. M. NMR structure of antibiotics plipastatins A and B from Bacillus subtilis inhibitors of phospholipase A(2). FEBS Lett. 485, 76–80 (2000). 11086169 10.1016/s0014-5793(00)02182-7

[bib50] Babasaki, K. , Takao, T. , Shimonishi, Y. & Kurahashi, K. Subtilosin A, a new antibiotic peptide produced by Bacillus subtilis 168: isolation, structural analysis, and biogenesis. J. Biochem. 98, 585–603 (1985). 3936839 10.1093/oxfordjournals.jbchem.a135315

[bib51] Mhammedi, A. , Peypoux, F. , Besson, F. & Michel, G. Bacillomycin F, a new antibiotic of iturin group: isolation and characterization. J. Antibiot. 35, 306–311 (1982). 6804427 10.7164/antibiotics.35.306

[bib52] Liu, W. T. et al. Imaging mass spectrometry of intraspecies metabolic exchange revealed the cannibalistic factors of Bacillus subtilis. Proc. Natl Acad. Sci. USA 107, 16286–16290 (2010). 20805502 10.1073/pnas.1008368107 PMC2941286

[bib53] Lamsa, A. , Liu, W. T. , Dorrestein, P. C. & Pogliano, K. The Bacillus subtilis cannibalism toxin SDP collapses the proton motive force and induces autolysis. Mol. Microbiol. 84, 486–500 (2012). 22469514 10.1111/j.1365-2958.2012.08038.x PMC3839633

[bib54] Yang, Y. et al. A plasmid-born Rap-Phr system regulates surfactin production, sporulation and genetic competence in the heterologous host, Bacillus subtilis OKB105. Appl. Microbiol. Biotechnol. 99, 7241–7252 (2015). 25921807 10.1007/s00253-015-6604-3

[bib55] Seyedsayamdost, M. R.. , Traxler, M. F. , Clardy, J. & Kolter, R. Old meets new: using interspecies interactions to detect secondary metabolite production in actinomycetes. Methods Enzymol. 517, 89–109 (2012). 23084935 10.1016/B978-0-12-404634-4.00005-X PMC4004031

[bib56] Shank, E. A. et al. Interspecies interactions that result in Bacillus subtilis forming biofilms are mediated mainly by members of its own genus. Proc. Natl Acad. Sci. USA 108, E1236–E1243 (2011). 22074846 10.1073/pnas.1103630108 PMC3228442

[bib57] Wilking, J. N. et al. Liquid transport facilitated by channels in Bacillus subtilis biofilms. Proc. Natl Acad. Sci. USA 110, 848–852 (2013). 23271809 10.1073/pnas.1216376110 PMC3549102

[bib58] Asally, M. et al. Localized cell death focuses mechanical forces during 3D patterning in a biofilm. Proc. Natl Acad. Sci. USA 109, 18891–18896 (2012). 23012477 10.1073/pnas.1212429109 PMC3503208

[bib59] Seydlová, G. et al. Surfactin production enhances the level of cardiolipin in the cytoplasmic membrane of Bacillus subtilis. Biochim. Biophys. Acta 1828, 2370–2378 (2013). 23845875 10.1016/j.bbamem.2013.06.032

[bib60] Gonzalez-Pastor, J. E. , Hobbs, E. C. & Losick, R. Cannibalism by sporulating bacteria. Science 301, 510–513 (2003). 12817086 10.1126/science.1086462

[bib61] Goessweiner-Mohr, N. , Arends, K. , Keller, W. & Grohmann, E. Conjugative type IV secretion systems in Gram-positive bacteria. Plasmid 70, 289–302 (2013). 24129002 10.1016/j.plasmid.2013.09.005 PMC3913187

[bib62] Kienesberger, S. et al. Interbacterial macromolecular transfer by the Campylobacter fetus subsp. venerealis type IV secretion system. J. Bacteriol. 193, 744–758 (2011). 21115658 10.1128/JB.00798-10 PMC3021226

[bib63] Boguslawski, K. M. , Hill, P. A. & Griffith, K. L. Novel mechanisms of controlling the activities of the transcription factors Spo0A and ComA by the plasmid-encoded quorum sensing regulators Rap60-Phr60 in Bacillus subtilis. Mol. Microbiol. 96, 325–348 (2015). 25598361 10.1111/mmi.12939 PMC4701059

[bib64] Branda, S. S. , Gonzalez-Pastor, J. E. , Ben-Yehuda, S. , Losick, R. & Kolter, R. Fruiting body formation by Bacillus subtilis. Proc. Natl Acad. Sci. USA 98, 11621–11626 (2001). 11572999 10.1073/pnas.191384198 PMC58779

[bib65] Sambrook, J. & Russell, D. W. (eds) Molecular cloning: a laboratory manual (Cold Spring Harbor Laboratory Press, 2001).

[bib66] Wach, A. PCR-synthesis of marker cassettes with long flanking homology regions for gene disruptions in S. cerevisiae. Yeast 12, 259–265 (1996). 8904338 10.1002/(SICI)1097-0061(19960315)12:3%3C259::AID-YEA901%3E3.0.CO;2-C

[bib67] Wilson, G. A. & Bott, K. F. Nutritional factors influencing the development of competence in the Bacillus subtilis transformation system. J. Bacteriol. 95, 1439–1449 (1968). 4967198 10.1128/jb.95.4.1439-1449.1968 PMC315105

[bib68] Shemesh, M. & Chai, Y. A combination of glycerol and manganese promotes biofilm formation in Bacillus subtilis via histidine kinase KinD signaling. J. Bacteriol. 195, 2747–2754 (2013). 23564171 10.1128/JB.00028-13 PMC3697245

