# Supplementary material for: Not so simple, not so subtle: the interspecies competition between Bacillus simplex and Bacillus subtilis and its impact on the evolution of biofilms
Source: NPJ Biofilms Microbiomes. 2016 Jan 27;2:15027–. doi: 10.1038/npjbiofilms.2015.27 (PMC5515258; doi:10.1038/npjbiofilms.2015.27)
Supplement: Supplementary Information [file npjbiofilms201527-s1.pdf]

Not so simple, not so subtle: The interspecies competition between *Bacillus simplex* and *Bacillus subtilis* and its impact on the evolution of biofilms

Gili Rosenberg[1], Nitai Steinberg[1,4], Yaara Oppenheimer-Shaanan[1,4], Tzvia Olender[1,4], , Shany Doron[1], Julius Ben Ari[3], Alexandra Sirota-Madi[2], Zohar Bloom-Ackermann[1] and Ilana Kolodkin-Gal[1*]

Supporting Information

<u>Supplementary information content:</u>

Supplementary Materials and Methods

Supplementary tables (S1-S2)

Supplementary references

Supplementary figures (S1-S16)

Supporting Materials and Methods

Selective Media preparation

Selective media were prepared in LB or LB-agar using antibiotics at the following final concentrations: 100 µg/ml ampicillin (AG Scientific), 10 µg/ml kanamycin (AG Scientific), 10 µg/ml chloramphenicol (Amresco), 10 µg/ml tetracycline (Amresco), 100 µg/ml spectinomycin (Tivan Biotech), and 1 µg/ml erythromycin (Amresco) + 25 µg/ml lincomycin (Sigma Aldrich) for MLS.

Interaction assay

To analyze the cell-number percentage of each bacterium during the interaction between *B. subtilis* and *B. simplex*, interaction plates were prepared as described, and incubated for the required time period. The interaction formation was then divided into three sections. The *B. subtilis* section (*B. sub*) consisted of the entire *B. subtilis* biofilm, not including the thick wrinkle that surrounded *B. simplex*. The interaction section (int) consisted the *B. subtilis* wrinkle and the *B. simplex* cells that were attached to it (detaching the wrinkle from the *B. simplex* colony was done simply by lifting it). The *B. simplex* section (*B. sim*) consisted of the entire *B. simplex* colony excluding the cells that were attached to the wrinkle. As controls, colonies of *B. subtilis* and *B. simplex* were grown alone and analyzed. Each section was harvested, inserted into 1.5 ml eppendorf tubes contained 200 µl PBS and mildly sonicated.

Sonication

Sonication was done using the BRANSON digital sonifier, model 250, with a thin needle. The biofilm was mildly sonicated (Amplitude -10%, pulse - 5 sec). The cell viability after sonication was tested by the analysis of the cells morphology (as described in section 4.8- Fluorescence microscopy) and by analysis of the replicating cells found in the culture prior to and following the sonication. Our analysis found that the described procedure had little or no effect on the morphology or viability of cells grown in *Bacillus* biofilms.

The cell-solutions were then diluted, plated on LB-agar plates, and incubated at 37˚C overnight to allow the formation of colonies. The colonies were differentiated by their morphology and color (*B. subtilis* flat and grayish colonies, *B. simplex* round sticking up milky color colonies) and counted for statistical analysis.

Supernatant production

A single *B. subtilis* WT or plasmid cured strain or plasmid cured mutants derived from the biofilm interspecies interaction colony, isolated on a solid LB plate, was inoculated into 3 ml of LB broth, grown overnight at room temperature and diluted to OD 1 Then either (i) 100µl of the overnight culture were inoculated into 100ml MSgg and grown in a 300 milliliter flask at 37°C with shaking for 2, 4, 6 and 8 hours as indicated in each corresponding figure legend or (ii) alternatively 20µl of the overnight culture were inoculated into 20ml B4 and grown in a 50 milliliter flask at 37°C with shaking for 8 hours. In both protocols, the conditioned medium was then centrifuged at 8000 rpm for 10 minutes; supernatant was removed and filtered through a 0.22 µm filter.

Assessing the effect of *B. subtilis* supernatant on *Bacillus* growth

Cells were grown in presence or absence of *B. subtilis* WT, or plasmid cured strain, or plasmid cured mutants derived from the biofilm interspecies interaction colony Supernatants as described at Supernatant production. Final concentration of 0.5X or 0.25X as indicated in each corresponding figure legend, while X represent the initial concentration of the molecule in the *B. subtilis* active supernatant.

Mass-spectrometry of surfactin

Accept for supplementary figure 7, we used an HPLC protocol optimized to detect and analyze surfactin isomers. The samples were analyzed on LC-MS system which consisted of Dionex Ultimate 3000 HPLC coupled to the LTQ Orbitrap Discovery hybrid FT mass spectrometer equipped with electrospray ionization source (Thermo Fisher Scientific Inc.). Chromatography separation parameters were as follow:

LTQ Orbitrap Discovery hybrid FT mass spectrometer equipped with electrospray ionization source (Thermo Fisher Scientific Inc.). Chromatography separation parameters were as follow:

Time, min	Solvent A,%<br>Water + 0.1% AcOH	Solvent B, %<br>Acetonitrile + 0.1% AcOH
0	85	15
2	85	15
14	4	96
20	4	96
20.1	85	15
24.5	85	15
Other Parameters		
Temperature of HPLC column, ^{0}C		40
Temperature of the sample tray, ^{0}C		15
Flow, µl/min		400
Volume of injection, µl		5

HPLC separations were carried out using Kinetex Hexyl-Phenyl column (2.1×150 mm, particle size 2.6 µm, Phenomenex). Mass spectrometer was operated in positive ionization mode, ion source parameters were as follows: spray voltage 3.5 kV, capillary temperature 300^{0}C, ion-transfer optics parameters were optimized using automatic tune option, sheath gas rate (arb) 35, and auxiliary gas rate (arb) 15. Mass spectra were acquired in the m/z 150-2000 Da range. The LC-MS system was controlled and data were analyzed using Chromeleon and Xcalibur software (Thermo Fisher Scientific Inc.). We used a standard obtained from Sigma is a mixture of surfactins. Peak areas of all detected surfactins were calculated and used for quantitative analysis.

For supplementary figure 7, we used LC-MS system which consisted of Thermo Scientific Accela HPLC coupled to the LTQ Orbitrap Discovery hybrid FT mass spectrometer equipped with electrospray ionization source (Thermo Fisher Scientific Inc.), For solvent A 25mM Ammonium acetate was used.

Purification of the cannibalism toxins and Mass-spectrometry of SdpC

A single *B. subtilis* WT colony, isolated on a solid LB plate, was inoculated into 3 ml of LB broth, grown overnight at room temperature. 100µl of the overnight culture were inoculated into 100ml MSgg and grown in a 300 liter flask at 37°C with shaking for 8 hours. The MSgg conditioned medium was centrifuged at 8000 rpm for 10 minutes removed and filtered through a 0.22 µm filter. 50ml of *B. subtilis* supernatant was enriched for proteins using a 3KDa Millipore Centricon and resuspended with 2ml PBS to a final concentration of 25X. The active proteins fraction filtered through a 0.22 µm filter and further analyzed using Mass Spectrometry.

Assessing the effect of surfactin enriched fractions on *Bacillus* growth

Cells were grown in presence or absence of *B. subtilis* small molecules fraction from the WT or *srfAA* mutant in final concentration of 4X, 2X, 1X or 0.1x indicated in each corresponding figure legend, while X represent the initial physiological concentration of the molecule in the *B. subtilis* active supernatant.

Assessing the effects of Proteins fractions on *Bacillus* growth

Cells were grown in presence or absence of *B. subtilis* proteins fraction from the WT or a double mutant for Δ*sdpC*Δ*skfA* in a final concentration of 5X, 2.5X, 1.25X or 0.31X indicated in each corresponding figure legend, while X represent the initial physiological concentration of the proteins in the *B. subtilis* active supernatant.

Testing the effects of *B. subtilis* bioactive fractions on biofilm development

To analyze the effects of the collected bioactive fractions on *B. simplex* biofilm formation, the methanol fractions that were eluted from C18 SPE column and the proteins fraction separated by 3KDa Millipore Centricon were added to MSgg-agar plates, in final concentration of 2X (fractions test activity) or 0.5X 0.625X (methanol fraction and proteins fraction concentrations in the synergism assay). The fractions activity was tested for indicated strains. 2 µl of *B. simplex* grown to mid-logarithmic phase was plated onto the treated plates and incubated at 30°C to the required time period

***RapP* complementation**

To generate the P*rapP-rapP phrP* complementation construct, we did as shown at McLoon paper [1]. a PCR product containing the *rapP phrP* coding region plus 500 bp of upstream sequence was amplified from *B. subtilis* 3610 DNA with the primer pair 349/350. sequence was cloned into SphI and SalI restriction sites of pDG1662 (laboratory collection). The destination vector, containing a polylinker and chloramphenicol resistance cassette between two arms of the *amyE* gene [2] was integrated into the *amyE* locus of PY79 by transformation. The integrated reporter was further introduced by transformation into 3610 plasmid cured strain.

Alignment of *B. subtilis* reads and identification of mutations

Sequencing reads of 101 nt were aligned separately for each sample to the reference genome of B. subtilis 3610 (Genbank: NZ_CM000488) that was downloaded from NCBI. The reads were aligned using Novoalign 2.08.01 (Novocraft Technologies Sdn Bhd, http://www.novocraft.com) with the default parameters and [-r Random]. Detection of mutations (mismatches and insertions) was done by comparing the alignments of each sample to the alignments of the ancestor B. subtilis sample that was also sequenced. Genomic positions that consistently differed between both alignments (>70%) were recorded as mutations. Genomic positions with no aligned reads, which had aligned reads in the ancestor sample, were recorded as deletions.

***B. simplex* genome assembly and mutation identification**

Next-generation sequencing of *B. simplex* yielded 5,312,308 single-end reads of 101 bp. Sequence adapters and low quality reads were removed using trimmomatic [3] and SGA. The reads were then assembled with Velveth [4] using an optimized kmer of 61 and a minimum contig length of 200, followed by Sequencher (GeneCodes Corp. minimum of 95% identity over 50bp). This yielded an assembly of 5.5Mb composed from 167 contigs with N50 of 17,084bp. RAST server (Luts et al., 1989) was further used to annotated 5714 proteins. To call variants from the mutants, their sequence reads were aligned to the *B. simplex* BA2H3 assembly with BWA [5], followed by removing of PCR duplicates with picard (The Picard toolkit http://broadinstitute.github.io/picard/). Freebayes (Garrison E, Marth G. Haplotype-based variant detection from short-read sequencing. *arXiv preprint arXiv:1207.3907 [q-bio.GN]* 2012) was apply to call variants using m=20 and F=0.65, and annovar for variant annotation.

Table 1: Strains list

Name	Genotype	Reference
IKbs1	NCIB3610 Wild Type	[6]
IKbs4 PY79	PY79	Laboratory collection
IKbs222	*Δhag:: tet*	Laboratory collection
IKbs225	*ΔcheA:: tet*	Laboratory collection
IKbs226	*ΔcheY:: tet*	Laboratory collection
IKbs 335	*ΔdegU:: tet*	Laboratory collection
IKbs70	*amyE::P_{hag} -gfp* (*cam*)	Laboratory collection
IKbs432	*ΔsdpC:: spec, ΔskfA:: mls*	This study
IKbs227	*ΔsrfAA:: mls*	Laboratory collection
IKbs526	*ΔsrfAA:: mls, ΔsdpC:: spec, ΔskfA:: kan*	This study
IKbs527	*ΔsdpC:: spec, ΔskfA:: mls amyE::$P_{hyperspank}$-gfp* (*cam*)	This study
IKbs528	*ΔsrfAA:: mls amyE:: $P_{hyperspank}$ -gfp* (*cam*)	This study

IKbs529	*ΔsrfAA:: mls, ΔsdpC:: spec,* *ΔskfA:: kan, amyE:: P* $_{hyperspank-}$ *gfp* (*cam*)	This study
IKbs2	Plasmid cured	Laboratory collection
IKbs530	*amyE::*P $_{rapP}$ *-rapP phrP* (*cam*)	This study
IKbs85	*amyE::P* $_{sdpA-}$ *-lux* (*cam*)	Laboratory collection
IKot9 *Bacillus simplex*	Wild-type	This study
IKot10 *Bacillus toyonensis* BCT-7112	Wild-type	This study
IKbs 584	Interaction evolved strain 1	This study
IKbs 585	Interaction evolved strain 2	This study
IKbs 586	Interaction evolved strain 3	This study
IKbs 587	Interaction evolved strain 4	This study
IKbs 588	Interaction evolved strain 5	This study

Table 2: Primers list

Name	Sequence (5'-3')	Purpose
skfkoA	ggtgcgttaggggttatgattgca	Deletion by LFH PCR
skfkoB	gagtctattgacatagctcccatgc gccgactgcgcaaaagacataat gccgactgcgcaaaagacataat	Deletion by LFH PCR
skfkoC	ctggcaaccctcaaaattgaatgcat ttgagaata ggg agt tga gcg tat ttgc	Deletion by LFH PCR
skfkoD	atgacgtgcttccctaagctgtatttg	Deletion by LFH PCR
sdpCKOA	ttaaccttagtggttatagggtggagacc	Deletion by LFH PCR
sdpCKOB	caattcgccctatagtgagtcgt caaattattatacctccattaagttatttctccattatct	Deletion by LFH PCR
sdpCKOC	ccagcttttgttccctttagtgagtccattataatt gagtgtcttgcggattgc	Deletion by LFH PCR
sdpCKOD	gactgcgggagatattgctgagc	Deletion by LFH PCR
16s27F	aaggaggtgwtccarcc	Amplification of ribosome 16S unit
16s1525R	agagtttgatcmtggctcag	Amplification of ribosome 16S unit
F16s start	gatgggagcttgctccctgat g	Sequencing of ribosome 16S unit
F16s middle	gctaacgcattaagcactccg cc	Sequencing of ribosome 16S unit
PrapPrapP_F	cccgcatgcttcatccggagactatttatgaacaa	Cloning of rapP gene to amyE on the bacterial chromosome
PrapPrapP_R1	cccgtcgacttaggtggtagcaccattcttgca	Cloning of rapP gene to amyE on the bacterial chromosome

Supplementary Figures

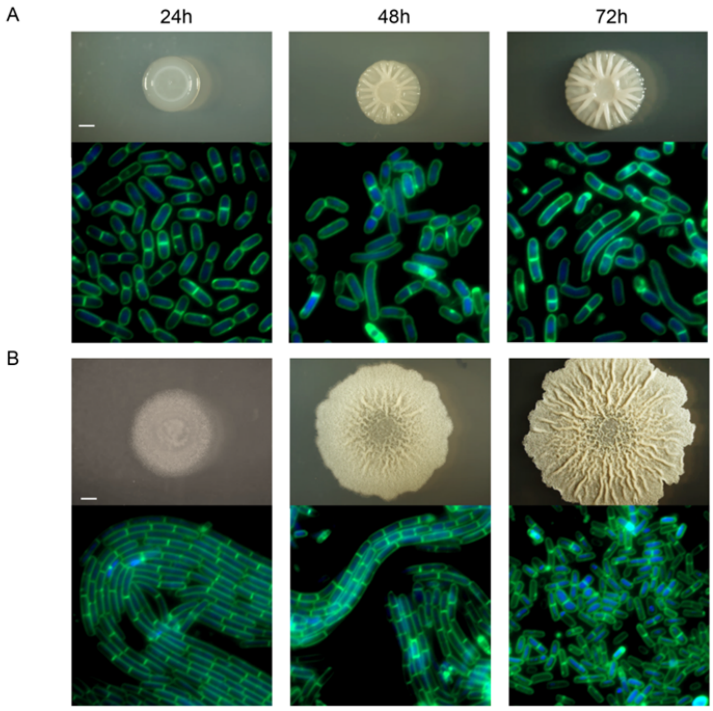

Supplementary figure 1| **The development of *B. subtilis* and *B. simplex* complex three dimensional colonies. A| Upper panel –** Top-down view of *B. simplex* colonies grown at 30°C on biofilm-inducing plates on days 1-3. Scale bar represents 2 µm. **Lower panel-** Fluorescent microscope images of *B. simplex* biofilm cells grown on biofilm-inducing plates stained with FM 1–43 (green), and DAPI (blue) on days 1-3. **B| Upper panel –** Top-down view of *B. subtilis* colonies grown at 30°C on biofilm-inducing plates on days 1-3. Scale bar represents 2 µm. **Lower panel-** Fluorescent microscope images of *B. subtilis* biofilm cells grown on biofilm-inducing plates stained with FM 1–43 (green), and DAPI (blue) on days 1-3.

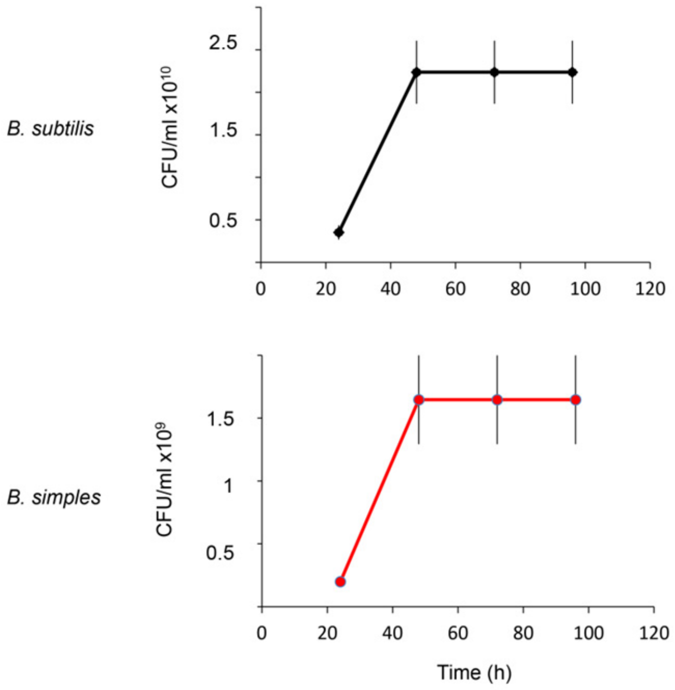

Supplementary figure 2| **Number of bacteria in the biofilms of *B. subtilis* and *B. simplex* during biofilm development when grown in isolation.** | Shown is number of *B. subtilis* (*upper*) and *B. simplex* (*lower*) Colony Forming Units (CFU) from biofilms grown separately at 30°C on MSgg biofilm-inducing medium. n=6, error bars represents the standard deviation.

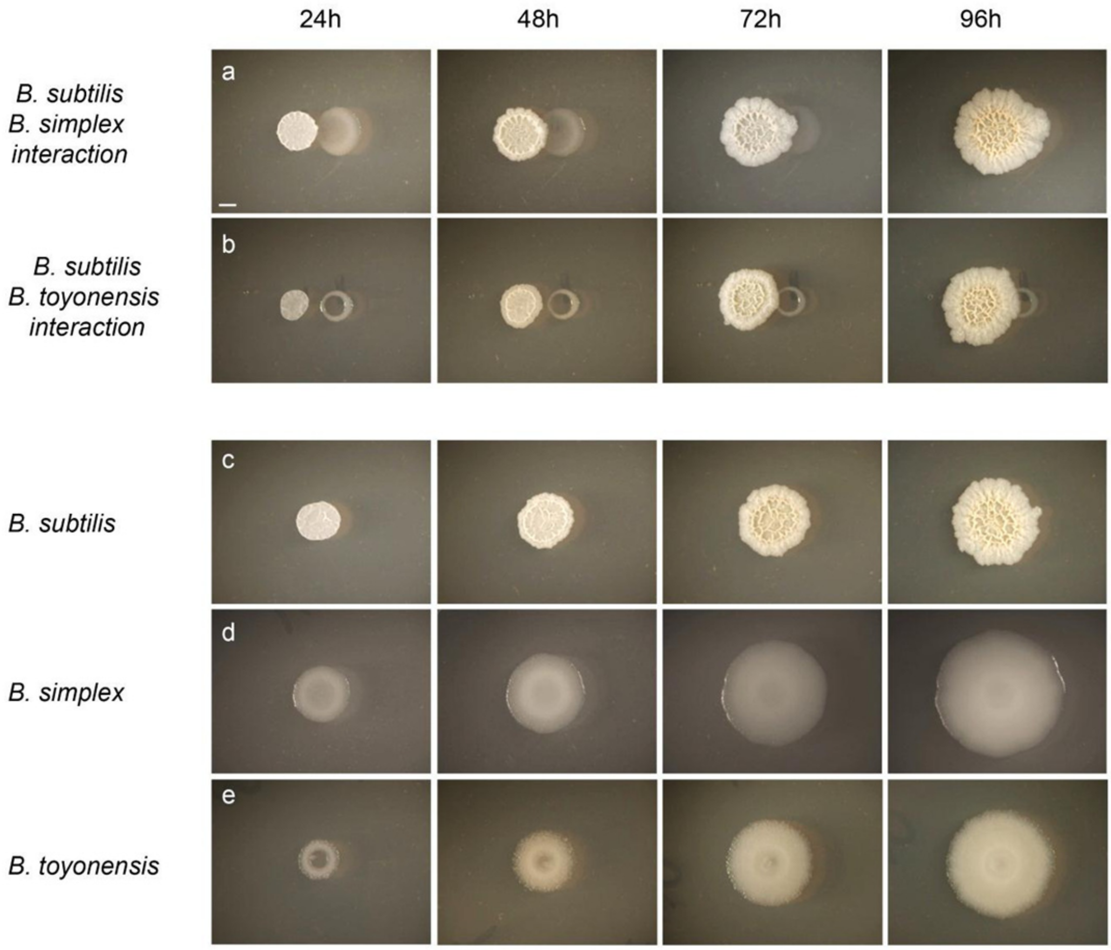

Supplementary figure 3| ***The interaction of B. subtilis* with competing *Bacillus spp.* biofilms.** Top-down view of colonies grown at 30°C on B4- rich biofilm-inducing plates, days 1-4 (a) Interaction between *B. subtilis* (left) and *B. simplex* (right), (b) Interaction between *B. subtilis* (left) and *B. toyonensis* (right), (c) *B. subtilis* grown separately, (d) *B. simplex* grown separately, (e) *B. toyonensis* grown separately, this control for *B. toyonensis* grown in isolation was also used for the independent biological repeat shown in Figure 1O . Scale bar represents 2 mm.

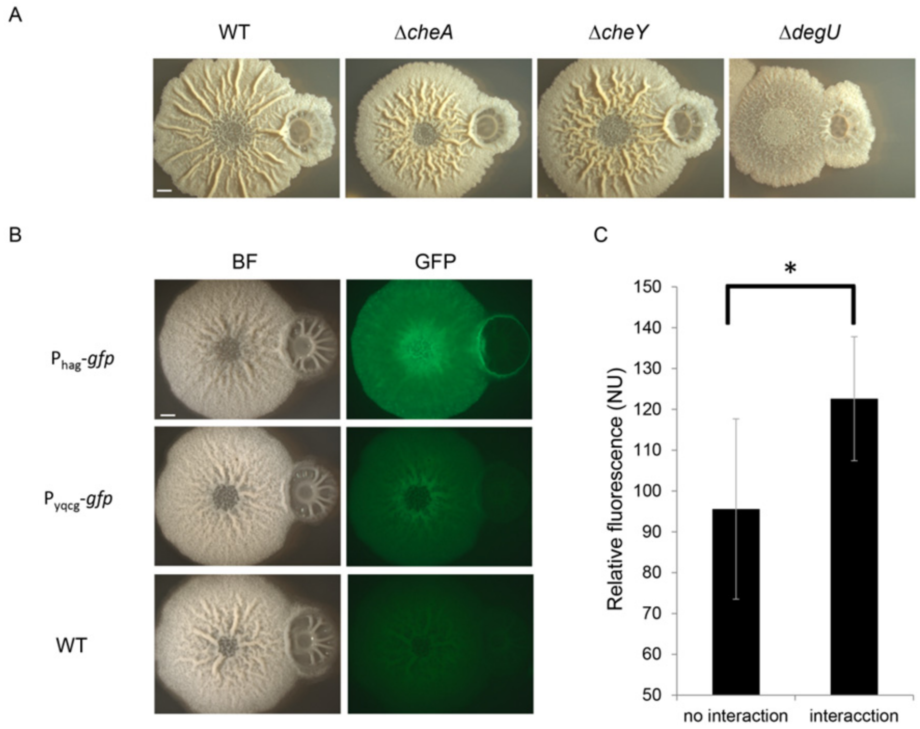

Supplementary figure 4| **The engulfment of *B. simplex* biofilm is mediated by *B. subtilis* motile subpopulation, and does not require chemotaxis. A|** WT and Δ*cheA,* Δ*cheY* and Δ*degU* mutants inoculated on MSgg plates at a distance of 0.6 cm from *B. simplex* biofilm engulf it within 3 days. Scale bar represents 2 mm. **B|** *B. subtilis* biofilms formed by strains harboring either a *gfp* fusion under the P_{hag} promoter or a *gfp* fusion under the P_{yqcG} promoter inoculated on MSgg at a distance of 0.8cm from a *B.simplex* biofilm grown for 2 days at 30ºC. Scale bars represent 2mm. **C|** Measurements of *hag* promoter activity in *B. subtilis* P_{hag}-*gfp* biofilm, inoculated on MSgg biofilm-inducing medium at a distance of 0.8cm from a *B.simplex* biofilm, grown for 2 days at 30ºC. The bacteria engulfing the *B. simplex* biofilm (interaction) and the bacteria in the *B. subtilis* edges of the biofilm opposites to the interaction side (no interaction) were mildly sonicated as described in the material and methods and was measured for *hag* expression. The GFP measurements were divided by OD Measurements. n=6, error bars represents the standard deviation. The difference between the pairs marked with an asterisk are statistically significant (*t* test, $p < 0.05$).

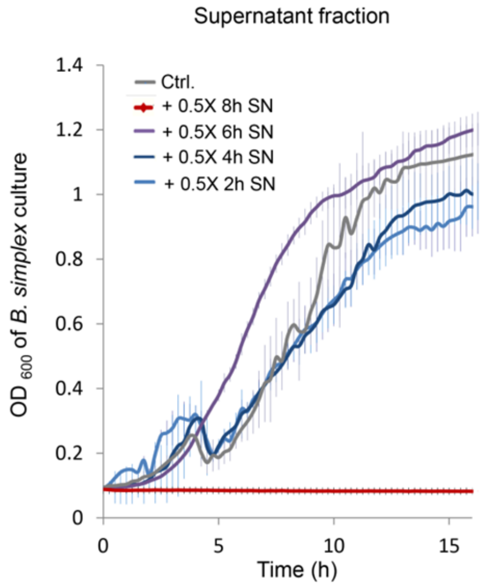

Supplementary figure 5| ***B. subtilis* secretes active molecules that inhibit *B. simplex* growth|** Growth curves of *B. simplex* cultured in liquid biofilm medium, media supplemented with 0.5X *B. subtilis* 2-8h supernatants and grown in 96-well plates, with shaking, at 30 °C. n=6, error bars represents the standard deviation.

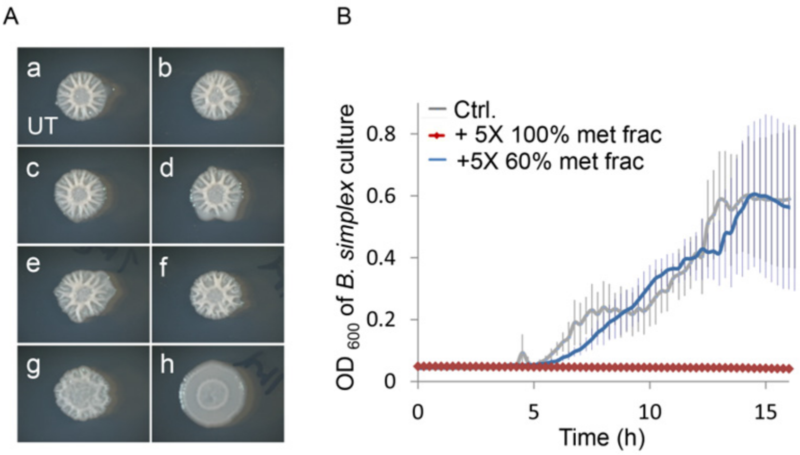

C

	Growth inhibition	Morphology changes
Untreated	/	/
60%	/	/
80%	++	+
100%	+++	++++

Supplementary figure 6| **The purification of the bioactive surfactin fraction. A|** Biofilm formation of *B. simplex* grown for 3 days on MSgg biofilm-inducing plates, media supplemented with 2X *B. subtilis* active supernatant that was purified on a C18 column using increasing methanol concentrations (a) Untreated. (b) Eluted with 10% methanol. (c) Eluted with 20% methanol. (d) Eluted with 30% methanol. (e) Eluted with 40% methanol. (f) Eluted with 60% methanol. (g) Eluted with 80% methanol. (g) Eluted with 80% methanol. (h) Eluted with 100% methanol. **B|** Growth curves of *B. simplex* in MSgg liquid biofilm-inducing media supplemented with 5X *B. subtilis* supernatant fraction eluted with 100% or 60% methanol. n=6, error bars represents the standard deviation.**C|** A summary of the activity of the 60%, 80% and 100% methanol fraction on *B. simplex* growth, four independent experiments.

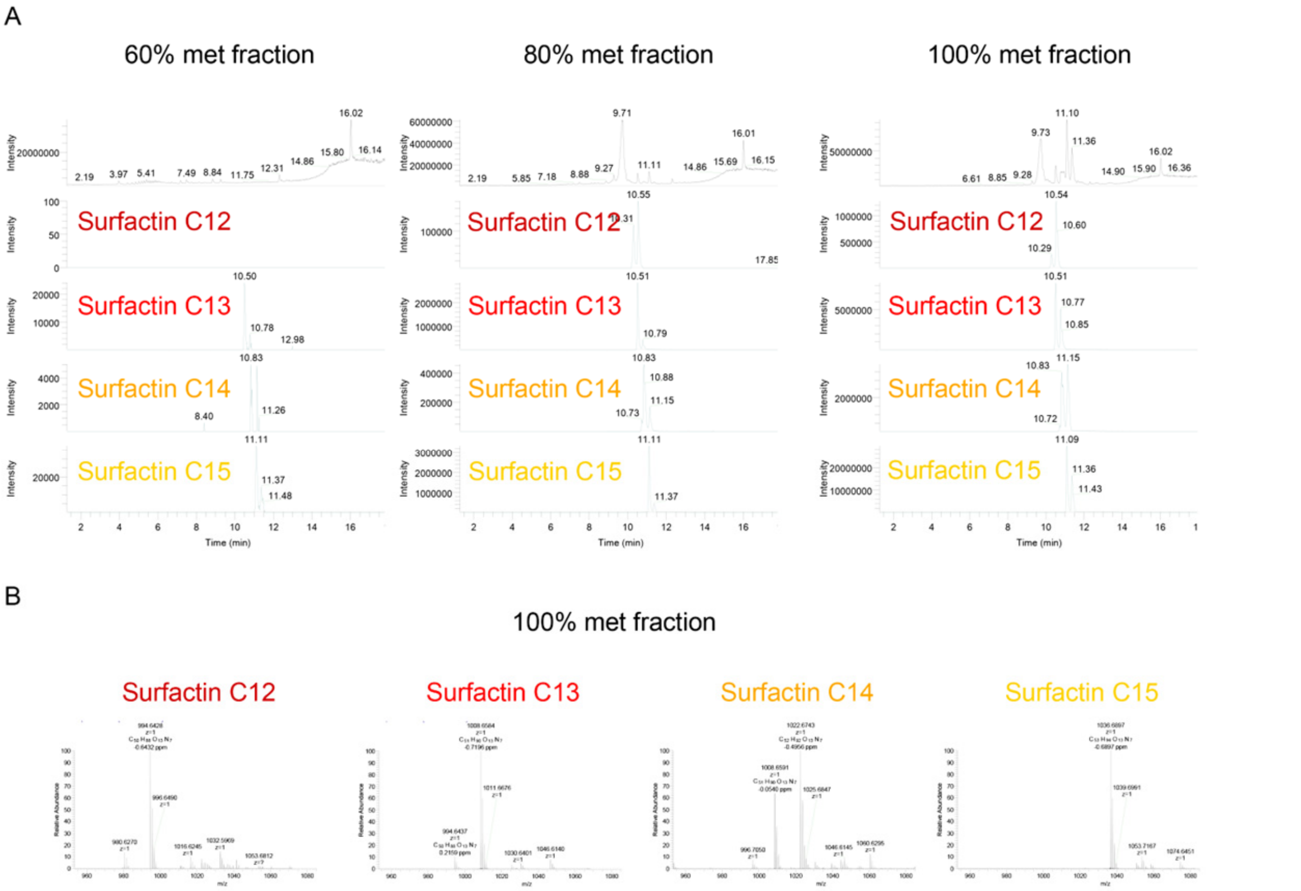

Supplementary figure 7| **The characterization of the active surfactin molecules in *B. subtilis* 100% methanol fraction. A|** LC-MS analysis of the 60%, 80% and 100% methanol fractions detected surfactin with varying carbon tail length surfactin lengths: C12 - 10.3, 10.5 min, surfactin C13-10.5, 10.8 min, surfactin C14-10.8, 11.1 min and surfactin C15 11.1, 11.4 min. The surfactin concentration decreased in positive correlation with the methanol concentration. LC-MS system which consisted of Thermo Scientific Accela HPLC coupled to the LTQ Orbitrap Discovery hybrid FT mass spectrometer equipped with electrospray ionization source (Thermo Fisher Scientific Inc.). **B|** Mass spectrometry of the different surfactin molecules in the active 100% methanol fraction

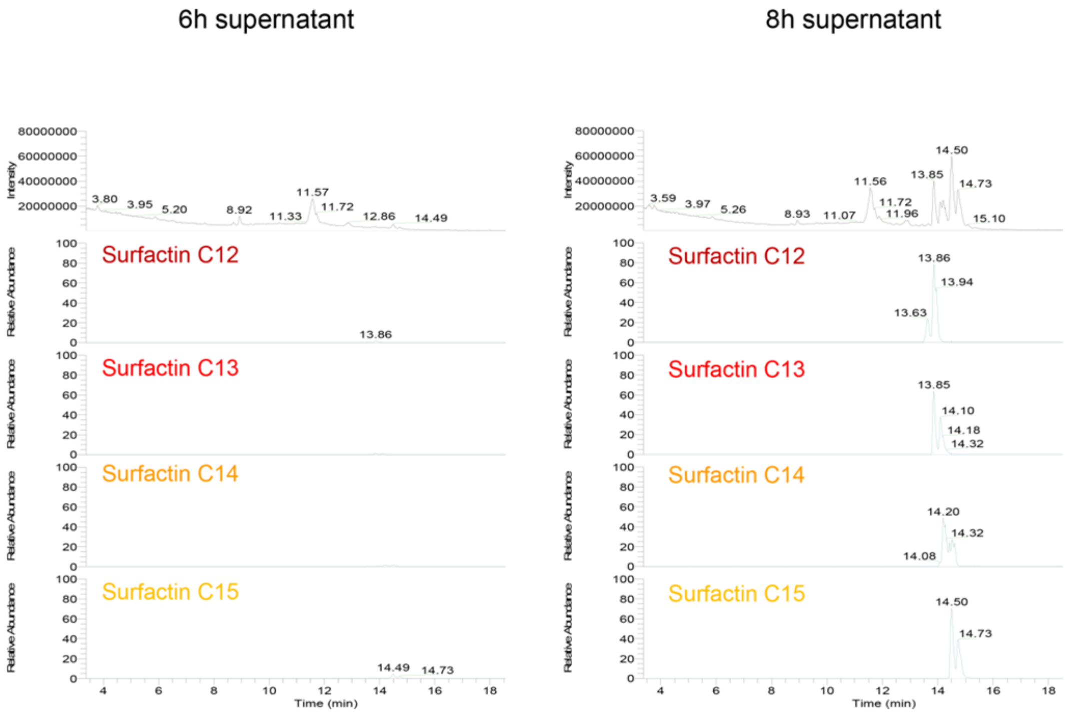

Supplementary figure 8| **LC-MS analysis of the varying tail length surfactin molecules that was found in the 8h active supernatant|.** LC-MS analysis of the *B. subtilis* non active supernatant obtained at 6 hours and the active supernatant obtained at 8 hours revealed surfactin isomers with varying carbon tail length only in the 8h supernatant. Samples were analyzed on LC-MS system which consisted of Dionex Ultimate 3000 HPLC coupled to the LTQ Orbitrap Discovery hybrid FT mass spectrometer equipped with electrospray ionization source (Thermo Fisher Scientific Inc.). Chromatography separation parameters were as described in Supporting Materials and Methods. HPLC separations were carried out using Kinetex Hexyl-Phenyl column (2.1×150 mm, particle size 2.6 µm, Phenomenex).

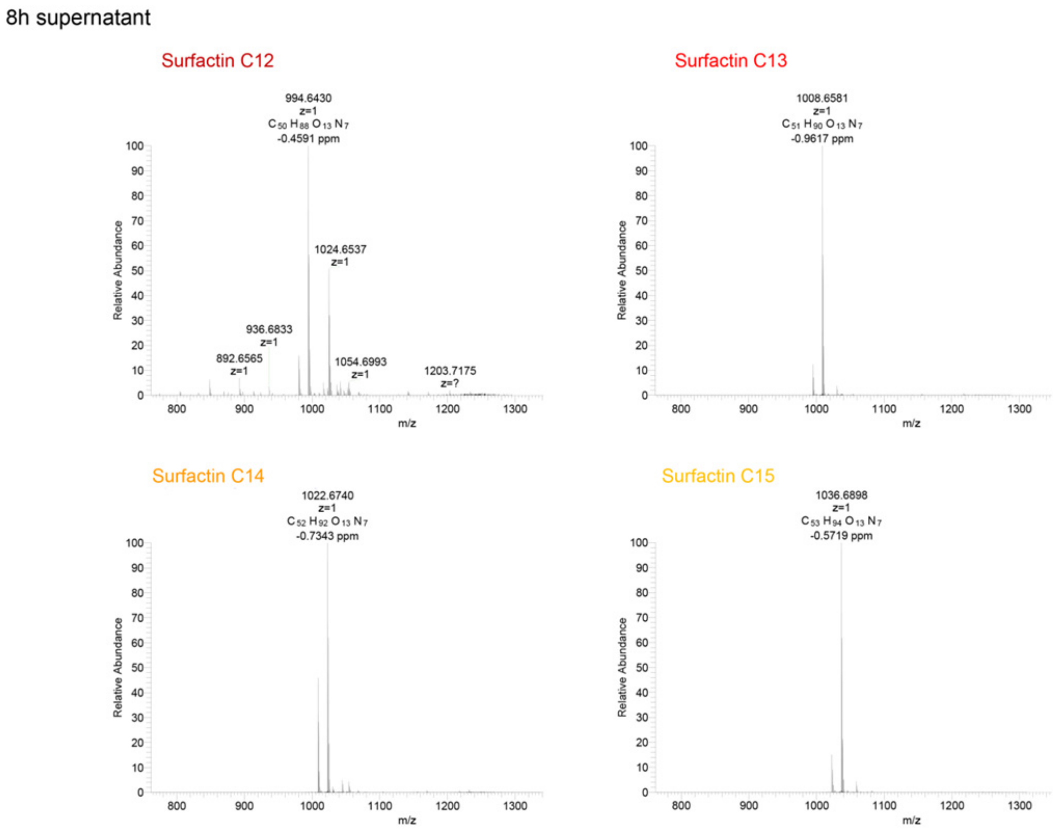

Supplementary figure 9| **Mass spectrometer analysis of the varying tail length surfactin molecules that was found in the 8h active supernatant.** C12- 994.642 g/mol, C13- 1008.657 g/mol, C14- 1022.673 g/mol, C15- 1036.688. Mass spectrometer was operated in positive ionization mode, ion source parameters were as follows: spray voltage 3.5 kV, capillary temperature 300^{0}C, ion-transfer optics parameters were optimized using automatic tune option, sheath gas rate (arb) 35, and auxiliary gas rate (arb) 15. Mass spectra were acquired in the m/z 150-2000 Da range. The LC-MS system was controlled and data were analyzed using Chromeleon and Xcalibur software (Thermo Fisher Scientific Inc.).

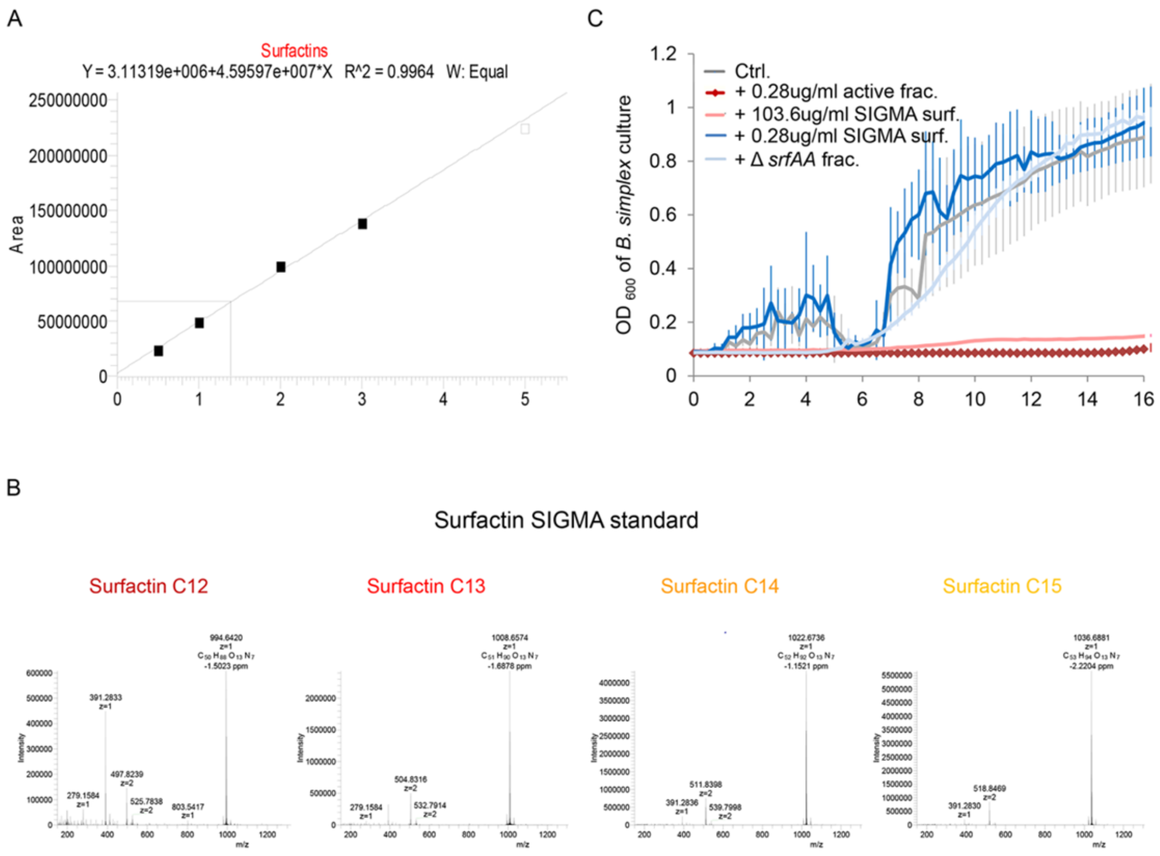

Supplementary figure 10| **Analysis of surfactants activity versus a commercial standard (Sigma)** Samples were analyzed on LC-MS system as described in Supporting Materials and Methods. Chromatography separation parameters were as described in Supporting Materials and Methods. **A|** Calibration Curve of commercial surfactin standard obtained from Sigma is a mixture of surfactins. Peak areas of all detected surfactins were calculated and used for quantitative analysis. Total concentration of surfactin detected in the sample using this analysis method was determined to be 1.4 µg/ml. x axis – concentration of all surfactin in µg/ml; y axis – sum of peak areas of detected surfactins (SIGMA S3523). **B|** Growth curves of *B. simplex* in 96-well plates with shaking in MSgg. Cells were supplemented with *B. subtilis* supernatant fraction or *B. subtilis srfAA* mutant supernatant fraction- in a volume equal to the WT fraction or with commercial surfactin (SIGMA) in different concentrations. n=4 wells, Error bars represent the standard deviation. **C|** Mass spectrometer analysis of the varying tail length surfactin molecules that was found in the commercially available surfactin (Sigma) performed as described in Supplementary Figure 9.

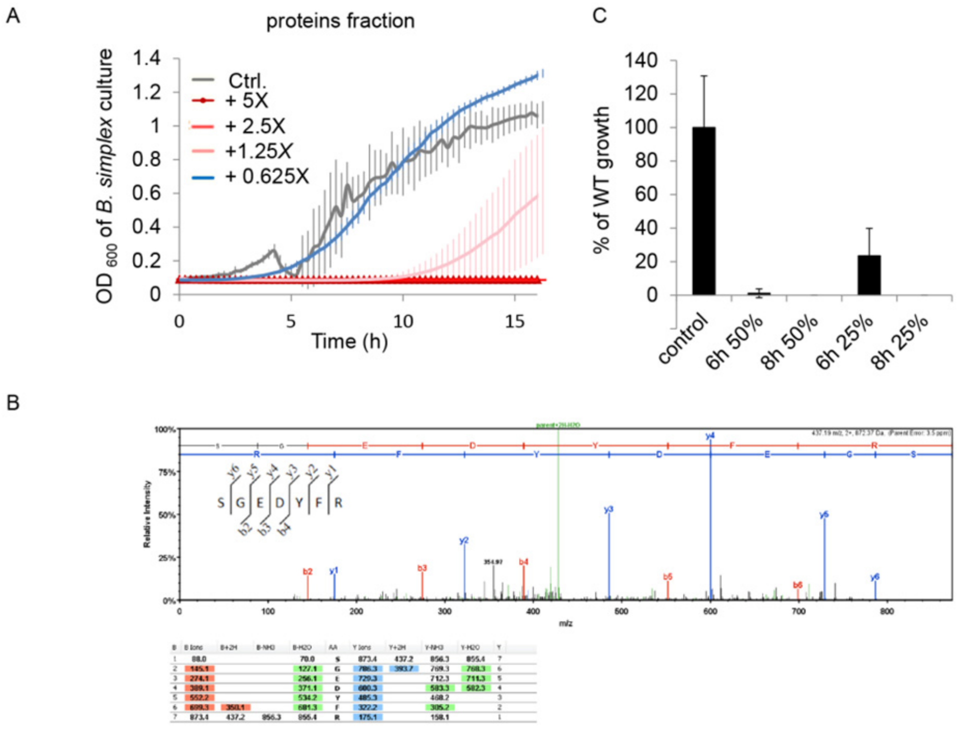

Supplementary figure 11| ***B. subtilis* secretes bioactive cannibalism toxins that inhibit *B. simplex* growth A|** Growth curves of *B. simplex* cultured in MSgg liquid biofilm medium supplemented with 1.25X, 2.5X or 5X *B. subtilis* supernatant protein fractions, enriched using a 3KDa Centricon, and grown in 96-well plates, with shaking, at 30 °C. n=6 error bars represents the standard deviation. **B|** Collision induced dissociation MS/MS spectrum of a single semi-tryptic peptide $_{39}$SGEDYFR$_{45}$ found in the protein Killing Factor SdpC. m/z denotes mass-to-charge ratio. b_n and y_n denote the N-terminal and C-terminal fragments, respectively. SdpC was the only peptide present in the purified fraction **C|** Relative growth of *B. simplex* for 10 hours compared with an untreated control grown with shaking in the presence of the indicated volume/volume ratios of *B. subtilis* supernatant protein fractions in MSgg medium.

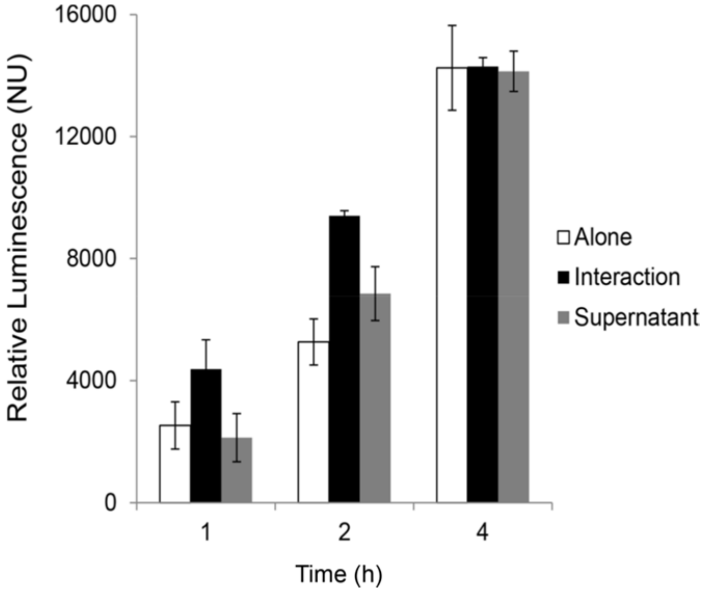

Supplementary figure 12| **Direct contact with *B. simplex* cells and to a lesser extent the supernatant enhance the expression of the *sdp* operon.** Data shown represent the transcription of *sdpA* using luciferase as reporter and the measured time course of normalized units of luminescence (luminescence divided by OD_{600}). Luminescence of a strain harboring *sacA::*P_{sdpA}*-luciferase* without (white) or in presence of supernatant of *B. simplex* (OD_{600}=0.5) culture diluted 1:200 (grey) or with a *B. simplex* (OD_{600}=0.5) culture diluted 1:2000 (black). Single colonies of the desired strains isolated over LB plates were grown to their mid-logarithmic phase of growth (4 hours at 37 °C with shaking). Starter cultures were diluted 1:200 for *B. subtilis* harboring *sacA::*P_{sdpA}*-luciferase* and in 3 ml liquid mLB biofilm medium and with *B. simplex* culture or supernatant as indicated . The cultures were grown for indicated times at 37 °C with shaking and the optical density at 600 nm and luminescence (sensitivity setting, 200) were measured every 2 hours min. In all relevant time points CFU were taken. Importantly in the interaction (black) the cultures were of vast majority of *B. subtilis* cells as they were mixed in 10:1 ratio with *B. simplex* and gradually eliminated the *B. simplex* cells. Averages and standard deviations of at least four independent experiments measured with three technical repeats are shown.

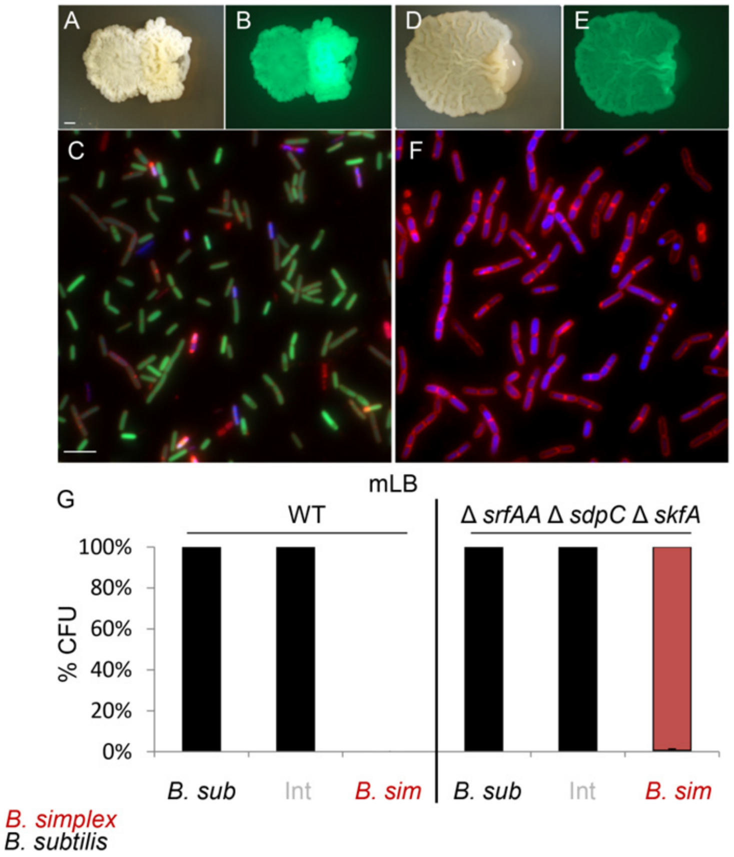

Supplementary figure 13| **A triple mutant in the production of surfactin, SdpC and SkfA shows reduced ability to eradicate *B. simplex* biofilms. A-F|** *B. simplex* biofilm were inoculated at a distance of 0.3cm from *B. subtilis* WT or Δ*sdpC,* Δ*skfA* and Δ*srfAA* triple mutant harboring the leaky $P_{hyperspank}$-*gfp* construct on mLB medium, for 2 days, separated and stained with FM 4–64 (red), and DAPI (blue). Scale bar for the interaction plates represents 2 mm, scale bar for the single-cell fluorescence microscope image represents 5µm. (A),(D) Bright field colonies images, (B),(E) GFP fluorescence images of the colonies in A and D images. (C),(F) fluorescence microscope image: green- *B. subtilis* strain expressing GFP, red-membrane stain and blue DAPI DNA stain were used to mark both the *B. subtilis* and *B. simplex.* **G|** Percentage of *B. subtilis* and *B. simplex* CFUs day 2 post-inoculation of *B. simplex* and *B. subtilis* WT or *B. subtilis* Δ*sdpC,* Δ*skfA,* Δ*srfAA* strains at distance of 0.3 cm, grown on mLB medium. The interacting colonies were divided into three sections: *B. sub* – *B. subtilis*, Int - interaction zone and *B. sim* – *B. simplex*. Each section was harvested, sonicated and plated to determine the number of replicative cells of each species. n=3 Error bars represents the standard deviation.

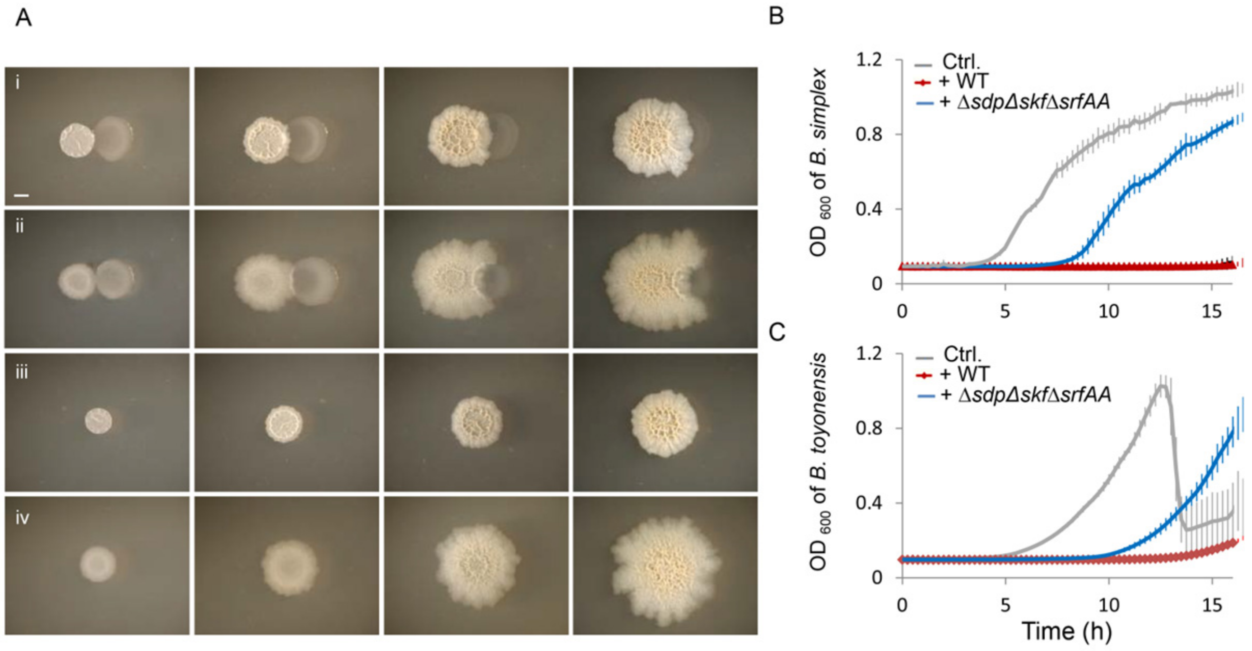

Supplementary figure 14| **A triple mutant in the production of Surfactin SdpC and SkfA null mutant shows reduce ability to eliminate rival *Bacillus* species. A |** *B. simplex* biofilms were inoculated at a distance of 0.3cm from *B. subtilis* WT or Δ*sdpC,* Δ*skfA* and Δ*srfAA* triple mutant strains, on B4-rich biofilm medium, for 1-4 days. (i) Interaction between *B. subtilis* (left) and *B. simplex* (right), (ii) interaction between *B. subtilis* Δ*sdpC,* Δ*skfA* and Δ*srfAA* triple mutant strains (left) and *B. simplex* (right), (iii) *B. subtilis* WT strain. (iv) *B. subtilis* Δ*sdpC,* Δ*skfA* and Δ*srfAA* triple mutant strains. **B|** Growth curves of *B. simplex* cultured in liquid B4-rich biofilm inducing medium supplemented with 0.01X *B. subtilis* B4- rich biofilm inducing medium supernatant of 8h growth from WT or Δ*sdpC*Δ*skfA* mutant strain cultures, were grown in 96-well plates, with shaking, at 30 °C. n=6, error bars represents the standard deviation. **C|** Growth curves of *B. toyonensis* cultured in liquid B4- rich biofilm inducing medium supplemented with 0.005X *B. subtilis* B4- rich biofilm inducing media supernatant of 8h growth from WT or Δ*sdpC*Δ*skfA* mutant strain cultures, were grown in 96-well plates, with shaking, at 30 °C. n=6 error bars represents the standard deviation.

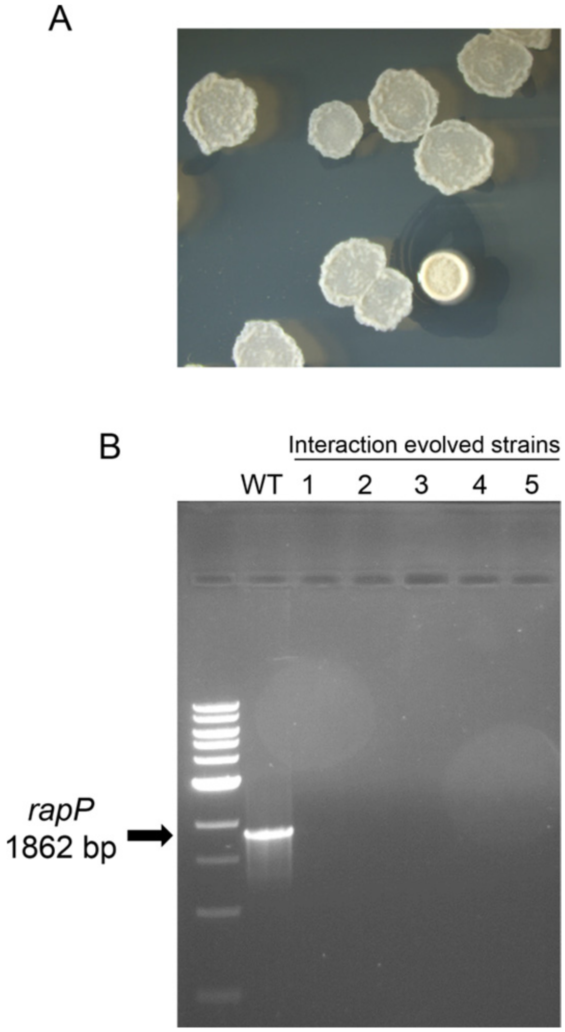

Supplementary figure 15| **Identification and conformation of bacterial strains from *B. subtilis* biofilm that loss their native plasmid. A|** The quantification of plasmid-cured cells was performed as described in materials and methods. Plasmid cured colonies were distinguished by their round and high colonies morphology and by their distinct yellowish color. **B|** Confirmation of the natural plasmid loss of the evolved strains was done by PCR analysis. The evolved strains showed lack of the *rapP* gene.

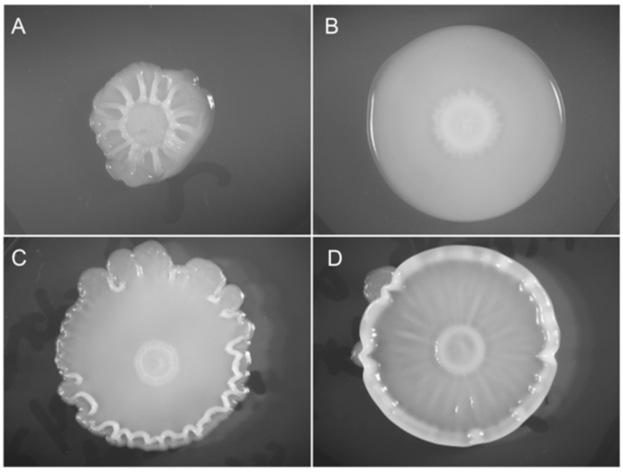

Analysis of *B. simplex* mutants

Mutant	Protein	Effect	Change
Mut 1	Spo0A	Nonsynonymous	G643A
Mut 2	Spo0A	Frameshift deletion	534_535del
Mut 3	Spo0A	Nonsynonymous	T193G
Mut 4	Tyrosine - protein kinase EpsD	Frameshift deletion	556_559del
Mut 5	UDP - glucose dehydrogenase	Frameshift deletion	918delA

Supplementary figure 16| **The mutagenesis pattern of *B. simplex* biofilm cells is affected by the interspecies interaction between the biofilms. A-D|** *B. simplex* mutants defected in biofilm formation. (A) WT (B) Flat biofilm mutagenesis pattern characterizing mutants derived from an interspecies interaction. (C) Flowery biofilm mutagenesis pattern characterized the mutants discovered in the *B. simplex* biofilm grown alone (D) Plate like structure characterized the mutants discovered in the *B. simplex* biofilm grown alone. **Table:** The summary of the genetic analysis of the *B. simplex* mutants forming a flat biofilm that survived the *B. subtilis*-*B. simplex* interaction.

Supplementary References

1. McLoon AL, Guttenplan SB, Kearns DB, Kolter R, Losick R. Tracing the domestication of a biofilm-forming bacterium. *Journal of bacteriology* **193**, 2027-2034 (2011).

2. Kolodkin-Gal I, Romero D, Cao S, Clardy J, Kolter R, Losick R. D-amino acids trigger biofilm disassembly. *Science* **328**, 627-629 (2010).

3. Luts A, Uddman R, Sundler F. Neuronal galanin is widely distributed in the chicken respiratory tract and coexists with multiple neuropeptides. *Cell and tissue research* **256**, 95-103 (1989).

4. Zerbino DR, Birney E. Velvet: algorithms for de novo short read assembly using de Bruijn graphs. *Genome research* **18**, 821-829 (2008).

5. Li H, Durbin R. Fast and accurate long-read alignment with Burrows-Wheeler transform. *Bioinformatics* **26**, 589-595 (2010).

6. Branda SS, Gonzalez-Pastor JE, Ben-Yehuda S, Losick R, Kolter R. Fruiting body formation by Bacillus subtilis. *Proceedings of the National Academy of Sciences of the United States of America* **98**, 11621-11626 (2001).
